# RNA-Targeting CRISPR/CasRx system relieves disease symptoms in Huntington’s disease models

**DOI:** 10.1186/s13024-024-00794-w

**Published:** 2025-01-13

**Authors:** Yingqi Lin, Caijuan Li, Yizhi Chen, Jiale Gao, Jiawei Li, Chunhui Huang, Zhaoming Liu, Wei Wang, Xiao Zheng, Xichen Song, Jianhao Wu, Jiaxi Wu, Oscar Junhong Luo, Zhuchi Tu, Shihua Li, Xiao-Jiang Li, Liangxue Lai, Sen Yan

**Affiliations:** 1 Guangdong Key Laboratory of Non-Human Primate Research, Key Laboratory of CNS Regeneration (Ministry of Education), School of Medicine, GHM Institute of CNS Regeneration, Jinan University, Guangzhou, 510632 China; 2 State Key Laboratory of Bioactive Molecules and Druggability Assessment, Jinan University, Guangzhou, 510632 China; 3 Department of Neurology, Faculty of Medical Science, Guangzhou Red Cross Hospital, Jinan University, Guangzhou, China; 4 China-New Zealand Joint Laboratory On Biomedicine and Health, CAS Key Laboratory of Regenerative Biology, Guangdong Provincial Key Laboratory of Stem Cell and Regenerative Medicine, Guangzhou, Institutes of Biomedicine and Health , Chinese Academy of Sciences, Guangzhou, China; 5 Department of Systems Biomedical Sciences, School of Medicine, Jinan University, Guangzhou, Guangdong 510632 China

**Keywords:** CRISPR/CasRx, Huntington's disease, *HTT*, HD-KI pig

## Abstract

**Background:**

HD is a devastating neurodegenerative disorder caused by the expansion of CAG repeats in the *HTT*. Silencing the expression of mutated proteins is a therapeutic direction to rescue HD patients, and recent advances in gene editing technology such as CRISPR/CasRx have opened up new avenues for therapeutic intervention.

**Methods:**

The CRISPR/CasRx system was employed to target human HTT exon 1, resulting in an efficient knockdown of *HTT* mRNA. This therapeutic effect was substantiated in various models: HEK 293 T cell, the HD 140Q-KI mouse, and the HD-KI pig model. The efficiency of the knockdown was analyzed through Western blot and RT-qPCR. Additionally, neuropathological changes were examined using Western blot, immunostaining, and RNA sequencing. The impact on motor abilities was assessed via behavioral experiments, providing a comprehensive evaluation of the treatment's effectiveness.

**Results:**

CRISPR/CasRx system can significantly reduce *HTT* mRNA levels across various models, including HEK 293 T cells, HD 140Q-KI mice at various disease stages, and HD-KI pigs, and resulted in decreased expression of mHTT. Utilizing the CRISPR/CasRx system to knock down *HTT* RNA has shown to ameliorate gliosis in HD 140Q-KI mice and delay neurodegeneration in HD pigs.

**Conclusions:**

These findings highlight the effectiveness of the RNA-targeting CRISPR/CasRx as a potential therapeutic strategy for HD. Furthermore, the success of this approach provides valuable insights and novel avenues for the treatment of other genetic disorders caused by gene mutations.

**Supplementary Information:**

The online version contains supplementary material available at 10.1186/s13024-024-00794-w.

## Background

Huntington's disease (HD) is a neurodegenerative disease caused by the abnormal expansion of CAG repeats in the Huntington (*HTT*) gene located on chromosome 4 [1–4]. Mutant huntingtin proteins accumulate in the central nervous system in an age-dependent manner [5], affecting the protein transport [6–10], and gene transcription [11, 12]. Similar to the disease process and pathological features of HD, a variety of neurodegenerative diseases, such as Alzheimer's disease (AD), Parkinson's disease (PD), and amyotrophic lateral sclerosis (ALS), show obvious age-dependence and have the corresponding accumulation of toxic proteins [13–18]. Among these diseases, HD is a dominant inherited disease caused by a single gene mutation. This distinctive feature positions HD as a promising avenue for exploring strategies aimed at inhibiting the toxic proteins and developing effective therapeutic interventions.

Although much is known about the pathogenesis of Huntington's disease, and various therapeutic strategies have been explored [19], there are still no effective treatment strategies available. In-depth exploration of polyQ diseases has unveiled a diverse range of detrimental impacts caused by expanded polyQ on various cellular functions. Consequently, a prevailing theory has emerged, suggesting that inhibiting the expression of expanded polyQ proteins could serve as a promising approach to effectively treat these diseases [20–26].

Several researchers have reported remarkable therapeutic effects of antisense oligonucleotide (ASO) on various disease models [27–30]. A clinical study revealed that intrathecal administration of antisense oligonucleotides (ASO) can decrease the levels of mutant huntingtin protein (mHTT) in the cerebrospinal fluid of Huntington's disease (HD) patients, and other studies also have demonstrated that silencing HTT mRNA can effectively rescue various animal models of HD [22, 26, 31, 32]. This suggests that reducing huntingtin expression at the RNA level is a very promising therapeutic approach. At present, it has been proved that the progression of HD related pathology is closely related to the accumulation of mHTT. In addition, preventing the formation of mHTT can alleviate related neuropathology and improve motor dysfunction, which is the key to the treatment of HD [33].

The use of CRISPR/Cas9 knockout of *HTT* to inhibit expanded polyQ protein expression proved to be successful in the treatment of various different HD mouse models [34–36]. Our recent study further demonstrated the efficacy of CRISPR/Cas9-mediated *HTT* knockout in the HD-KI pig model [37]. These results suggest that knockout of *HTT* at the DNA level can be a therapeutic option for HD. However, it is important to acknowledge the potential risks of off-target effects associated with CRISPR/Cas9 system, as it can permanently and irreversibly alter the genome [38]. These risks should not be overlooked, particularly in clinical applications. The large size of the Cas9 protein greatly restricts the packaging and infection efficiency of AAV viruses, as well as the effectiveness of gene therapy. As a novel RNA interference tool, CRISPR/Cas13 is composed of a guide RNA (gRNA) and a Cas13 nuclease [39]. The Cas13 nuclease from Ruminococcus flavefaciens strain XPD3002 (CasRx) is more active, specific, and has a smaller molecular weight compared to other Cas13 nucleases, consisting of only about 930 amino acids [39]. The small size, which allows CasRx to be easily packaged into adeno-associated virus (AAV), and the low neurotoxicity of CasRx in mammals make CRISPR/CasRx an ideal and compatible system for efficient delivery in mammals [39, 40]. Moreover, in contrast to Cas9, CasRx does not possess a protospacer adjacent motif (PAM) limitation. This allows CasRx to process CRISPR arrays and cleave target RNAs with high efficiency and specificity, independently of PAM requirements [39]. The CasRx protein forms a complex with the gRNA, then the CasRx-crRNA complex binds to the target RNA, the CasRx endonuclease domain cleaves the RNA at specific sites, causing its degradation [39]. Therefore, using the CRISPR/CasRx system for RNA targeting appears to be a safer and viable alternative to gene therapy based on the DNA-targeting CRISPR/Cas9 system [41–44].

However, the efficacy of utilizing CasRx to mitigate neurodegenerative effects in patients still needs to be validated. Considering that larger mammals share similarities with humans in terms of brain size, development, and brain structures [45, 46], it is crucial to assess the safety of CasRx through preclinical studies conducted on larger animal models that closely resemble humans in biological and physiological aspects. Significant advancements have been achieved in establishing large animal models of Huntington's disease, including sheep [47–49], minipigs [50, 51], and macaques [52, 53], and these large animal models have proved to be valuable to the Huntington's disease research community with their unique advantages. Among them, our previously constructed the huntingtin knock-in (HD-KI) pig model was constructed by replacing the sequence in the corresponding site of the pig HTT with the human mutant HTT exon1 carrying abnormal expanded CAG repeat sequence, and model serves as an ideal animal model for studying therapeutic interventions in HD, as it faithfully recapitulates the pathological features and motor deficits observed in HD patients [50]. It is essential for conducting preclinical evaluations using these large animal models that closely resemble humans in terms of biological and physiological characteristics [54].

The validity of the CasRx system in large animal model function-restoration study remains unknown. At present, there is still a lack of studies investigating the use of the CRISPR/CasRx to silence HTT for the treatment of HD at different disease stages [44, 55]. In order to explore the therapeutic potential of CRISPR/CasRx in HD, we designed gRNAs targeting the human *HTT* exon1. HD 140Q-KI mice at different disease stages [56], and HD-KI pigs [50] were used to test the efficiency of *HTT* mRNA knockdown and the associated neuropathological changes. Our findings demonstrate that the stereotaxic injection of CRISPR/CasRx into the striatum of HD 140Q-KI mice, both in the early and late stages of the disease, effectively downregulates the expression of *HTT* mRNA and alleviates the neuropathology associated with gliosis. Furthermore, the stereotactic injection of CRISPR/CasRx successfully mitigated some neurodegeneration and partially normalized gene expression dysregulation in HD-KI pigs. These results provide compelling evidence for the therapeutic potential of CRISPR/CasRx in the treatment of HD. The promising therapeutic outcomes of CasRx in large animal models of neurodegenerative diseases underscore the significant potential of CasRx for treating neurological disorders in humans.

## Methods

### Plasmid construction

The plasmid, HTT-N171-150Q contains huntingtin N-terminal 1–171 amino acid sequences, including 150 polyglutamine repeats. And the sequence was cloned into pRK5 vector, and then expresses under the control of CMV promoter. To clone the AAV plasmid AAV-CAG-CasRx, CasRx was PCR-amplified from pXR001, the plasmid encoding CasRx (Addgene, 109049). SgRNAs oligonucleotides targeting *HTT* mRNA were constructed using online software and chemically synthesized by IGE Biotechenology (Guangzhou China). The sequence of gRNAs was: HTT gRNA1 AGGCCTTCATCAGCTTTTCCAGGGTCGCCA; HTT gRNA2 GGTCGGTGCA GCGGCTCCTCAGCCACAGCC; HTT gRNA3 GCTGAGGAAGCTGAGGAGGCG.

GCGGCGGCG; HTT gRNA4 GTGCCTGCGGCGGCGGCTGAGGAAGCTGAG; and Ctrl gRNA CGGAATTCATCCAGCCACCAGGGTCGCCG. More detailed sequences of the gRNAs were shown in Supplementary Table 1. And they were cloned into pXR003 (Addgene, 109053).

### Cell culture and transfections

Human embryonic kidney (HEK) 293 T cells in growth medium composed of Dulbecco's Modified Eagle Medium (DMEM) supplemented with 10% (v/v) fetal bovine serum (FBS; Thermo Fisher Scientific) and 1% (v/v) penicillin/streptomycin solution (Thermo Fisher Scientific). The cells were maintained at 37ºC in a humidified 5% CO_2_ atmosphere. To assess the ability of CRISPR/CasRx to clear mutant huntingtin aggregates in vitro, the 293 T cells were seeded onto 24-well plates at an average density of 2 × 10^5^ cells per well and then transfected. The 293 T cells were divided into four groups, control gRNA, HTT gRNA1 + 2, HTT gRNA3 + 4, HTT gRNA1 + 2 & 3 + 4 groups. All four groups were co-transfected with HTT N171-150Q and AAV-CAG-CasRx, and the corresponding gRNA plasmids were also co-transfected. The 293 T cells for RT-qPCR and WB assays were divided into the same four groups and co-transfected with AAV-CAG-CasRx and the corresponding gRNA plasmid. Transfection of the above plasmids were performed using Lipofectamine 3000 (Thermo Fisher Scientific) according to the manufacturer's instructions. After 48 h of transfection, the cells were collected for western blotting, immunofluorescence, and RT-qPCR.

### Immunohistochemistry

After removing the culture medium, the cells were fixed with cold 4% paraformaldehyde for 10 min at room temperature, then washed three times with PBS, and subsequently blocked with a solution containing 2% goat serum and 0.1% TrionX-100 in 3% BSA for 1 h at room temperature. Following the blocking step, the cells were incubated with primary antibodies overnight at 4 °C. After the primary antibodies were removed, the cells underwent three washes with PBS. Subsequently, the cells were incubated with secondary antibodies at room temperature for a duration of 30 min. Fluorescent images were captured using a Zeiss microscope (Axiovert 200 MOT) equipped with a digital camera (Hamamatsu Orca-100) and Openlab software (Improvision Inc.).

The animals were anesthetized with isoflurane and transcardially perfused with 0.9% saline. The brains were then dissected, and one half of each brain was fixed in 4% paraformaldehyde in PBS at 4 °C for 24 h. Subsequently, the fixed brain tissue was dehydrated in 30% sucrose at 4 °C. After dehydration, the brains were embedded in tissue cryoprotectant (OCT) and sectioned into 30-μm coronal sections using a cryostat (Leica CM1950). For immunofluorescent staining, the brain slices were mounted onto glass slides that had been pre-coated. The following fixation and antibody incubation steps were consistent with the immunostaining steps described above in cells. For DAB staining, we used the Avidin–Biotin Complex kit (Vector ABC Elite, Burlingame, CA, USA). Imaging acquisition was performed using a confocal imaging system (Olympus FV3000 Microscope, Japan) or TissueFAXS PLUS (TissueGnostics, Vienna, AUT). All image analyses were conducted using the ImageJ software.

The following primary antibodies were used: mouse anti-NeuN (1:500; Millipore, MAB377), rat anti-GFAP (1:1000; Invitrogen, 13–0300), rabbit anti-Iba1 (1:500; WAKO, 019–1974), rabbit anti-GFP (1:1000; Invitrogen, A-11122), mouse anti-mEM48 (1:50; Millipore, MAB5374). The following secondary antibodies were used: goat anti-rat IgG Alexa Fluor 594 (1:1000; Invitrogen, A-11007), donkey anti-rabbit IgG Alexa Fluor 488 (1:1000; Abcam, ab150073), goat anti-mouse IgG Alexa Fluor 594 (1:1000; Abcam, ab150116). The antibodies used in the experiments were listed in detail in Supplementary Table 3.

### RT-qPCR analysis

Quantitative reverse transcription polymerase chain reaction (RT-qPCR) was employed to assess the mRNA expression levels of HTT. Total RNAs from 293 T cells, striatum of mice, and striatum of pigs were extracted using the Trizol method (Thermo Fisher Scientific, MA, USA). The extracted RNAs were assessed for purity and integrity before converting to complementary DNA (cDNA) with the PrimeScript™ RT Kit with gDNA Eraser (Takara, Kyoto, Japan). cDNAs were employed as templates for PCR, with the glyceraldehyde-3-phosphate dehydrogenase (GAPDH) serving as the internal reference. PCR primers for GAPDH and HTT were designed for amplification using TB Green Premix Ex Taq II (Takara, Kyoto, Japan). The CFX Connect Real-Time PCR Detection system (Bio-Rad, California, USA) was employed for PCR product detection, with each biological replicate were tested in technical triplicate. The relative expression levels of the genes were calculated using the cycle threshold (2^−ΔΔCt^) method. The formula used for this calculation was △Cq = Cq target gene—Cq internal reference gene, and △△Cq = △Cq experimental group—△Cq control group. The primer sequences used for the RT-qPCR analysis can be found in Supplementary Table 1.

### Western blot analysis

For western blotting analysis, the harvested cells and brain tissues from mice and pigs were grinded by Luka Grinding instrument (LUKYM-II, China). The homogenized samples were then lysed in ice-cold RIPA buffer (50 mM Tris, pH 8.0, 150 mM NaCl, 1 mM EDTA pH 8.0, 1 mM EGTA pH 8.0, 0.1% SDS, 0.5% DOC, and 1% Triton X-100) supplemented with Halt Protease Inhibitor cocktail (Thermo Scientific), 50 mmol/L NaF, and PMSF.The cells and tissue lysates were incubated at 4 °C for 60 min with gentle rocking. After incubation, the samples were sonicated and centrifuged at 12,000 rpm for 10 min. The resulting supernatant containing the protein was collected and the protein concentration was determined by using the BCA Protein Assay Kit (Solarbio). The equal amount of supernatant denatured protein was electrophoresed by SDS–polyacrylamide gel electrophoresis and then electrophoretically transferred to a nitrocellulose (NC) membrane in transfer buffer (20 mM tris–HCl, 150 mM glycine, and 20% (v/v) methanol). To prevent non-specific binding, the NC membrane was blocked with 5% milk/TBST (20 mM Tris–HCl, 150 mM NaCl pH 7.4 with 0.1% Tween 20) for 1 h at room temperature. Primary antibodies were diluted in a solution of 3% BSA/TBST and subsequently incubated with the NC membrane overnight at a temperature of 4 °C. After incubation with primary antibodies, the blotted membrane was washed three times with TBST for 10 min each. Subsequently, the membrane was incubated with horseradish peroxidase (HRP)-conjugated secondary antibodies in 5% milk/TBST for 1 h at room temperature. After undergoing three washes with TBST, the western blot images were developed using enhanced chemiluminescence (ECL) and captured using a ChemiScop 6000 instrument (CLiNqinxiang, Shanghai, China). The intensity of the protein bands was quantified using ImageJ Software (Bio-Rad) and subsequently normalized to the reference protein present in each lane.

The following primary antibodies were used: rabbit anti-vinculin (1:1000; Abcam, ab91459), mouse anti-NeuN (1:500; Millipore, MAB377), rat anti-GFAP (1:1000; Invitrogen, 13–0300), rabbit anti-Iba1 (1:500; WAKO, 019–1974), mouse anti-mEM48 (1:50; Millipore, MAB5374), mouse anti-1C2 (1:1000; Millipore, MAB1574), mouse anti-Flag (1:1000; Millipore, F1804). The following secondary antibodies were used: donkey anti-mouse (1:5000; Jackson ImmunoResearch, 715–035–151), donkey anti-rabbit (1:5000; Jackson ImmunoResearch, 711–035–152), goat anti-rat (1:5000; Invitrogen, 31,470). The antibodies used in the experiments were listed in detail in Supplementary Table 3.

### AAV production and purification

The AAV9-CasRx, AAV9-HTT gRNA1 + 2, AAV9-HTT gRNA3 + 4, and AAV9-Ctrl gRNA vectors were sent to PackGene Biotech for viral packaging and production (PackGene, Guangzhou, China). CasRx was packaged into one AAV, while HTT gRNA1 and 2, and HTT gRNA3 and 4 were packaged into two distinct AAVs. The scrambled sgRNA was used as control gRNA. During the packaging process, all the viral vectors were packaged into the AAV9. Following packaging and purification, the resulting AAV9 viral particles were stored in small aliquots at a temperature of −80℃ in a freeze. The company provided the titers of all the viruses, which were reported to be 1X10^13^ vector genomes (vg)/ml.

### Animals and ethics statement

All mouse procedures conducted in this study were granted ethical approval by the Institutional Animal Care and Use Committee of Jinan University (Approval No.: IACUC-20221118–02). The HD 140Q-KI mice, which express full-length mHTT containing human exon 1 with 140 CAGs [56], were obtained from Jackson Laboratory (#027409). Heterozygous 140Q-KI mice were generated through the breeding of male heterozygous mice with female wild-type C57BL/6 J mice. Mice were housed in the Division of Animal Resources at Jinan University, following a 12-h light/dark cycle. In the experiment, we used mice with an equal ratio of males to females. All procedures and husbandry practices were conducted in accordance with the guidelines outlined in the NIH Guideline for the Care and Use of Laboratory Animals. The use of Rongshui and Bama miniature pigs in this study, was in compliance with the Institutional of Animal Care and Use Committees (IACUC) of Guangzhou Institute of Biomedicine and Health (GIBH), Chinese Academy of Sciences (Animal Welfare Assurance # N2019083). The HD-KI male and female pigs carrying a mutant HTT allele with 150 CAG repeats from the previous study were selected as parents to produce offspring [50]. Wild type pigs were selected from the same litter. All pigs were used in this study were bred at the animal facility of Guangzhou Institute of Biomedicine and Health (GIBH), Chinese Academy of Sciences. To ensure the safety of personnel and animal welfare, the study adhered strictly to the guidelines stipulated in the ‘‘Guide for the Care and Use of Laboratory Animals (2011)’’.

### Genotyping

To genotype the HD-KI DNA fragment, specific primers were designed to amplify genomic DNA fragment containing the homologous arm and CAG repeats in *HTT*. Using primer Genotyping-mouse F (5 ’- ACTGCTAAGTGGCGCCGCGTAG—3’) and Genotyping-mouse R (5 ’- GAGGCAGCAGCGGCTGTGCCTG—3’) to produce PCR products for the identification of HD 140Q -KI mice. The PCR products for the identification of HD-KI pigs were generated using Genotyping-pig F (5’- GGAGAGCTGGGAGAGAATGCCAGTGTGACAGT −3’) and Genotyping-pig R (5’- GCGGCTGAGGCAGCAGCGGCTGTGCCTG −3’). The PCR conditions used were as follows: an initial denaturation step at 94℃ for 5 min, followed by 35 cycles of denaturation at 94℃ for 30 s, annealing at 65℃ for 30 s, and extension at 72℃ for 1 min and 30 s. A final extension step at 72℃ for 5 min was performed, and the samples were then held at 12℃ before being removed from the PCR machine. The template DNA was the genomic DNA isolated from the mouse tail and pig ear.

### Stereotaxic injection of AAV9

Following the protocol previously described by our laboratory [57], for the stereotaxic injection into the brain, HD 140Q-KI mice and control mice were anesthetized using isoflurane inhalation. All mouse procedures were approved by the Institutional Animal Care and Use Committee of Jinan University and conducted in strict adherence to the guidelines outlined in the National Institutes of Health Guide for the Care and Use of Laboratory Animals. The HD 140Q-KI mice were injected with AAV9-CasRx and AAV9-Ctrl gRNA as control group (Untreated), AAV9-CasRx, AAV9-HTT gRNA1 + 2, and AAV9-HTT gRNA3 + 4 as the treatment group (Treated), and the WT mice were injected with AAV9-Ctrl gRNA as the WT control group (WT Ctrl). The experiment was performed on the left and right hemispheres of other WT mice by injecting 2ul of AAV9-Ctrl gRNA on one side and the same volume of PBS on the other side. Viruses expressing gRNAs and CasRx were mixed at a ratio of 1:2 (Ctrl gRNA: CasRx = 1: 2; HTT gRNA1 + 2: HTT gRNA3 + 4: CasRx = 1: 1: 4), and 4 μl of the mixed viruses (a total of 4X10^10^ vg) were injected into both sides (2 μl each side) of the striatum. The mouse's head was securely positioned in a Kopf stereotaxic frame (Model 1900) equipped with a digital manipulator, a UMP3–1 Ultra pump, and a 10 μl Hamilton microsyringe (Hamilton Co., Reno, NV, USA). Based on the following coordinates (relative to the bregma) adjusted to the flat skull position: anteroposterior (AP), + 0.55 mm; mediolateral (ML), ± 2 mm; dorsoventral (DV), − 3.5 mm, then small holes were drilled in the skull with a drill. Virus injection was performed after a 33G needle was carefully inserted through the small hole for 3.5 mm. The microinjections were conducted at a rate of 0.20 μl/min, with the microsyringe being left in position for an additional 10 min after each injection. The mice were euthanized one and a half months after the virus injection.

Pigs also were anesthetized using isoflurane via inhalation. Following the procedures previously described by our previous research [37], when fully anesthetized, each pig was secured in a stereotaxic frame (RWD Instruments). All surgical procedures were conducted in compliance with the guidelines for the Care and Use of Laboratory Animals and biosafety protocols at the Guangzhou Institute of Biomedicine and Health, Chinese Academy of Sciences. The three-month-old pigs were divided into different groups for the injection of viruses. The WT control group (WT Ctrl) received injections of AAV9-Ctrl gRNA, and the HD control group (Untreated) received injections of AAV9-CasRx and AAV9-Ctrl gRNA. The HD treatment group (Treated) was injected with AAV9-CasRx, AAV9-HTT gRNA1 + 2, and AAV9-HTT gRNA3 + 4 at a ratio of 4: 1: 1. A total of 30 μl of the mixed viruses, corresponding to 3X10^11^ vg, were injected into both sides of the pig striatum, with 15 μl being delivered to each side. The injection coordinates were adjusted to the flat skull position: 5 mm posterior to bregma, 10 mm lateral to the midline (left or right side), and 30 mm ventral to the dura surface. Small holes were drilled in the skull using a drill, and a 26-gauge Hamilton syringe connected to a syringe infusion pump (World Precision Instruments, Inc., Sarasota, Florida, USA) was used to deliver the virus at a rate of 800 nl/min. In order to alleviate pain, meloxicam (2 mg/kg) was administered as an analgesic. Additionally, the pigs were placed on a warm pad to aid in their recovery from anesthesia.

### Behavioural analysis

All behavioral experiments were performed by blinded investigators. The behavior tests were performed using 12 mice per group and a assessed according to the method in our previous study [58]. Five weeks after the injection of the virus, motor coordination of HD 140Q-KI mice was assessed using a rotarod apparatus (Rotamex 4/8, Columbus Instruments International). Prior to actual testing, the mice were trained on the rotarod at 40 RPM for 5 min per day for three consecutive days to familiarize them with the task. During the testing phase, the speed of the rotarod was gradually increased to 40 RPM over a period of 5 min. The latency to fall from the rotarod was recorded for each trial. Each mouse underwent three trials, and the average data each mouse was calculated as an evaluation metric. The pole-climbing test was conducted to evaluate the mouse's movement and coordination abilities. For this test, a custom-made wooden rod measuring approximately 50 cm in length and 1 cm in diameter was used. The rod was wrapped with gauze to increase friction. The mouse was positioned in a face-down manner on the top of the vertical pole, and the time taken for it to descend from the top to the bottom platform was recorded. Before the actual testing, each mouse underwent a three-day training period to familiarize themselves with the task. The experimental procedure was repeated three times and the average descent time for each mouse was calculated as an evaluation metric. The balance beam task involves placing the mice on an elevated and narrow beam measuring 1 cm in width and 100 cm in length. The time taken by the mouse to cross the balance beam is recorded as the evaluation measure. Each mouse underwent three trials of the balance beam task, and the mean of the three pass times was taken.

The motor ability of the HD-KI pigs was assessed as previously described [37, 50]. The treadmill running test was conducted three months after the brain-injections of the viruses. For evaluate gait, the footprint tracking method was used. HD KI pigs were trained to traverse an 80 cm wide and 4.5 m long sandy pathway, and their footprints were recorded using a camera camcorder. To conduct the treadmill test, the pigs were positioned on a treadmill equipped with a closed cage to evaluate their running capacity. The closed cage facilitated the pig's movement on the conveyor belt. Prior to testing, the pigs underwent a treadmill training regimen for three consecutive days. During the test, the treadmill speed was maintained at 1.5 km/h.

### Electron microscopy analysis

Electron microscopy (EM) analysis of the pig striatum was conducted by BioServices (Servicebio, Wuhan, China). Freshly isolated pig brain tissue blocks were fixed with a solution containing 4% paraformaldehyde and 0.2% glutaraldehyde for a duration of 48 h. The fixed tissue blocks were then sectioned using a vibratome. To prepare the sections for electron microscopy, a series of dehydration steps were performed using increasing concentrations of ethanol and propylene oxide/Eponate 12 (1:1). Subsequently, the sections were embedded in Eponate 12. Ultrathin sections with a thickness of 60 nm were obtained using a Leica Ultracut S ultramicrotome. These thin sections were then counterstained with a 5% aqueous uranyl acetate solution for 5 min, followed by Reynolds lead citrate staining for another 5 min.

### RNA-seq and data analysis

The total RNA from the striatum of mice and pigs was isolated using RNAiso Plus (TaKaRa, Japan). Subsequently, the mouse RNA samples were sent to SequMed Bio Technology (Guangzhou, China), and the pig RNA samples were sent to HeQin Biotechnology Corporation (Guangzhou, China), then analyzed using the Illumina HiSeq X platform (Illumina, San Diego, CA, USA) for RNA-seq analysis and database construction. For each sample, 2 μg of RNA was used for the analysis. The raw RNA-sequencing data were aligned and quantified using STAR, followed by further analysis [59]. Genes with the *P*-value < 0.01 and an absolute fold change > 2 were considered as differentially expressed genes (DEGs) by using EdgeR [60]. To determine the functional enrichment of the DEGs, GO enrichment analysis was performed using TBtools and ClusterProfiler [61–64]. GO terms with a *P*-value < 0.01 and a hit rate > 0.05 were considered to be significantly enriched.

### Statistical analysis

When comparing two groups, a two-tailed Student t-test was employed to determine the statistical significance. For the analysis of multiple groups, one-way ANOVA was used to assess statistical significance. The data are presented as mean ± standard error of the mean (SEM). The quantification of Western blots was conducted using Image J software. All calculations were performed using GraphPad Prism 8 software. A *P*-value of less than 0.05 was considered statistically significant.

## Results

### CRISPR/CasRx efficiently reduces HTT

**Fig. 1:**
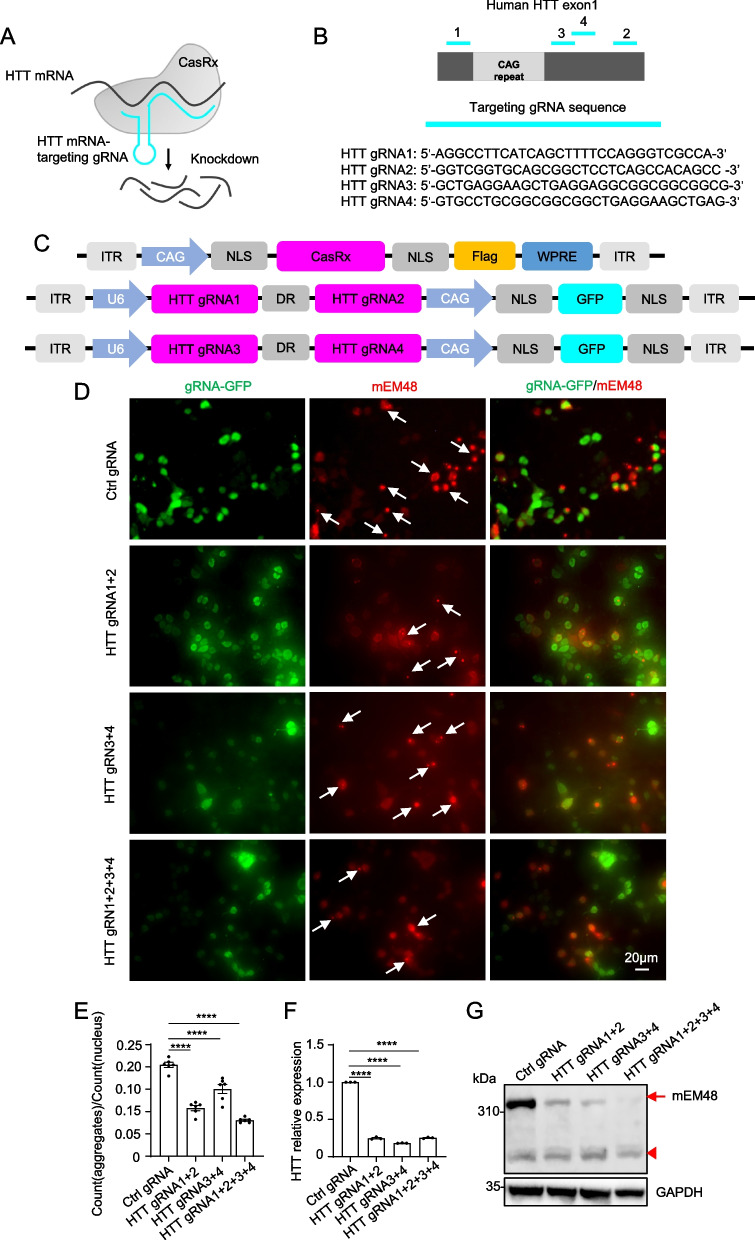
Reduce the expression of HTT in vitro using CRISPR/CasRx. **A** Schematic diagram of CasRx-mediated knockdown of HTT mRNA. **B** Schematic of the human HTT exon1 mRNA and the locations of 4 gRNAs (HTT gRNA1, HTT gRNA2, HTT gRNA3, HTT gRNA4) targeting sites (light blue bars), and below are 4 gRNAs target sequences. **C** The construct used to express CasRx under the control of the CAG promoter, the construct also expresses flag tag as a reporter. Two vectors used to express HTT gRNAs were constructed, and the HTT gRNAs (HTT gRNA1 and HTT gRNA2, HTT gRNA3 and HTT gRNA4) driven by the U6 promoters. The two vectors express green fluorescent protein (GFP) as a reporter. **D** Immunofluorescent staining analysis of the amounts of aggregates from HEK 293 T cells transfected with HTT-N171-150Q, CAG-CasRx and the candidate gRNAs. Scale bar: 20 μm. **E** Quantification of the aggregates in co-transfected HEK 293 T cells (*n* = 6 images per group). Data were analyzed by one-way ANOVA and presented as mean ± SEM. ****P < 0.0001. **F** Detection of HTT mRNA knockdown efficiency of different gRNAs in HEK 293 T cells transfected with CAG-CasRx and the candidate gRNAs using RT-qPCR. Data were analyzed by one-way ANOVA and presented as mean ± SEM. ****P < 0.0001. **G** Western blot analysis of full-length HTT from protein lysates isolated from HEK 293 T cells transfected with CAG-CasRx and the candidate gRNAs using mEM48 antibody. The full-length HTT was indicated by an arrow. Arrowheads indicate non-specific bands. GAPDH was used as a loading control

We first attempted to use the CRISPR/CasRx system to target and knock down HTT in cultured cells and screen for efficient gRNAs. While the Huntingtin protein (HTT) plays a crucial role in early development in mice [65–69], and complete elimination of HTT leads to the development of motor impairments in mice [70, 71]. However, several studies have demonstrated that long-term elimination of HTT in the striatum of adult mice [27, 34, 36, 68], pigs [26, 37], and rhesus monkeys [24, 27, 72, 73] is well tolerated. Given the more severe effects of mHTT and its influence on various downstream pathways, reducing the expression of the HTT through gene silencing still is a promising strategy [74]. Moreover, CRISPR/CasRx targeting is more inclined towards inducing a knockdown effect rather than achieving complete depletion. To this end, we designed four gRNAs containing 30 nucleotides to target the mature human *HTT* exon1 mRNA, both upstream and downstream of the CAG repeat expansion (Fig. 1A, B; Extended Data Fig. 1A). Compared with native Cas13d, Cas13d variants with a nuclear localization signal (NLS) sequence have higher efficiency of targeting RNA [39]. In order to improve efficiency and facilitate the detection of Cas13d variants (also called as CasRx), we used Cas13d variant with NLS at both the N- and C-terminals, and a Flag tag at the C-terminals. gRNA1 & gRNA2 and gRNA3 & gRNA4 targeting HTT were designed on the same vector, respectively (Fig. 1C).

To evaluate the efficiency of HTT gRNAs in reducing the number of aggregates, we transfected 293 T cells with CasRx, gRNAs (HTT gRNAs or non-targeting control gRNA (Ctrl gRNA)), and N-terminal mutant *HTT* fragments (HTT-N171-150Q), and then performed immunofluorescent staining to quantify the number of aggregates. The antibody mEM48 specifically labeled for N-terminal fragments of human HTT was used in the experiment [5]. Our results demonstrate that both HTT gRNA1 and 2, as well as HTT gRNA3 and 4, effectively reduced the number of aggregates. Notably, the combined use of all four HTT gRNAs resulted in the most significant reduction, with a decrease of approximately 50% (Fig. 1D, E). As all four HTT gRNAs target the mature human HTT exon 1 mRNA (Fig. 1B), we assessed the expression levels of HTT using RT-qPCR and western blot analysis. Our findings demonstrate that the CRISPR/CasRx system effectively silenced HTT expression at both the RNA and protein levels (Fig. 1F, G). Consistent with the reduction in aggregate numbers observed above, the use of all four HTT gRNAs in combination resulted in the most significant decrease in HTT. Consequently, we selected these four gRNAs for further investigation.

### CasRx silencing of HTT rescues mouse models of HD at different stages of disease

**Fig. 2:**
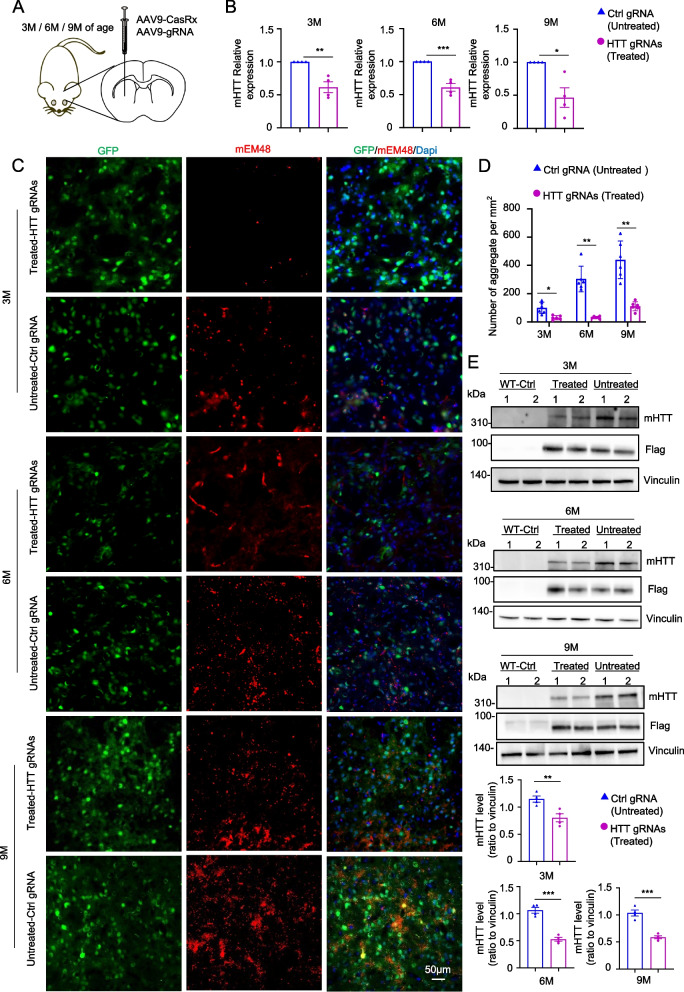
Therapeutic efficacy of CRISPR/CasRx in HD KI-140Q mice with different degrees of disease. **A** Schematic illustration of injecting AAV9-CasRx and AAV9-gRNA into the striatum of HD KI-140Q mice of different ages. **B** RT-qPCR analysis of mutant *HTT* mRNA expression in HD KI-140Q mice treated at 3, 6, and 9 months of age. *n* = 4 mice per group. Data were analyzed by unpaired two-tailed t-test and presented as mean ± SEM. **P = 0.0032, ***P = 0.0005, *P = 0.0109. **C** Representative immunofluorescent images of the striatum from HD KI-140Q mice injected with the CasRx/gRNA at 3, 6 and 9 months old. Antibodies for mEM48 and GFP (gRNA) were used. DAPI is used for nuclear staining. Scale bars: 50 μm. **D** Quantification of mHTT aggregates in HD KI-140Q mice treated at different ages. Cells expressing mHTT aggregates were counted per 0.1 mm.^2^, *n* = 6 mice per group. Data were analyzed by two-way ANOVA and presented as mean ± SEM. *P (3 M) = 0.0193, **P (6 M) = 0.0020, **P (9 M) = 0.0042. **E** Representative Western blots of mHTT expression in HD KI-140Q mice treated at 3, 6, and 9 months of age. Antibody used was the polyQ specific antibody 1C2. Flag indicated CasRx expression (CAG-CasRx constructs containing flag tag) and vinculin was used as a loading control. Beneath the blots, the quantification ratios of mHTT to vinculin were presented. Unpaired two-tailed t-test was used for statistical analysis of two groups and data were expressed as mean ± SEM. Four animals were used for Western blotting in each group: **P = 0.0098 (3 M), ***P = 0.0001 (6 M), ***P = 0.0003 (9 M)

HD is a typical neurodegenerative disease characterized by motor dysfunction and severe neuropathology, so the efficacy of treatment is closely related to the stage of the disease [4, 75]. Therefore, we conducted experiments to investigate the effect of CasRx-mediated silencing of HTT on HD 140Q-KI mice at different stages of the disease. The HD 140Q-KI mouse model expresses full-length mHTT with 140Q, as exon 1 of human *HTT* with 140 CAG repeats replaces exon 1 of endogenous mouse *HTT* [56]. We injected the heterozygous HD 140Q-KI mice at 3, 6, and 9 months of age (Fig. 2A), as the motor deficits in HD 140Q-KI mice are age-dependent and do not appear at 3 months of age. Dyskinesias were observed at 6 months of age, and marked motor impairments were evident at 9 months of age. Additionally, HD 140Q-KI mice began to form mHTT intranuclear deposits at 2 months of age, with the size and number of mHTT aggregates increasing with age [56, 76, 77]. AAV vector-encoded CasRx and HTT gRNAs or non-targeting control gRNA were used for stereotaxic injection into the bilateral striatum of mice, and six weeks after the injection, the mice were euthanized for subsequent analysis.

Consistent with the expression of the CRISPR/CasRx system in 293 T cells, the RT-qPCR results showed that the injection of AAV-CasRx/HTT gRNAs reduced *HTT* mRNA in HD 140Q-KI mice of different ages (Fig. 2B). Furthermore, it was observed that AAV-CasRx/HTT gRNAs efficiently silenced the expression of HTT in the striatum of different age groups, as demonstrated by immunofluorescence staining (Fig. 2C, D). Western blotting analysis using the 1C2 antibody, which reacts only with polyQ expanded protein [78, 79], revealed that the expression of CasRx/HTT gRNAs significantly reduced mHTT in the striatum of mice at different ages, compared to the CasRx/Ctrl gRNA -injected striatum (Fig. 2E). The results of western blotting analysis using the EM48 antibody were consistent with those of the 1C2 antibody, and a significant reduction in aggregates was observed after AAV-CasRx/HTT gRNAs injection, especially in 9-month-old treated HD 140Q-KI mice (Extended Data Fig. 2A). In summary, our findings demonstrate that the CRISPR/CasRx system can efficiently silence the expression of *HTT* at the RNA and protein levels in the striatum of HD 140Q-KI mice.

### CRISPR/CasRx treatment mitigated a portion of the neuropathological changes in the striatum of HD 140Q-KI mice

**Fig. 3:**
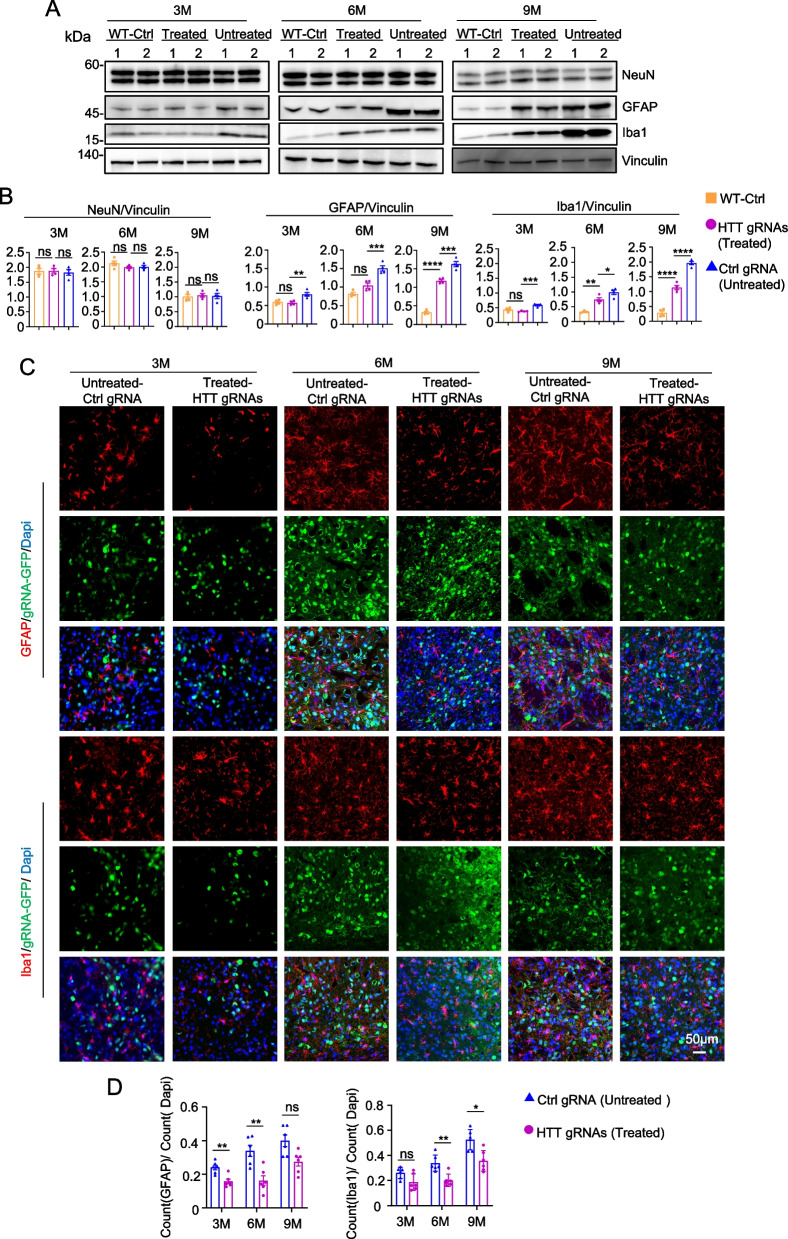
Analysis of the neuropathology in HD KI-140Q mice after AAV-CRISPR/CasRx treatment. **A** Western blots of neuronal (NeuN) and glial (GFAP, Iba1) proteins expression in the striatum of HD KI-140Q mice injected with AAV-CasRx/Ctrl gRNA (Untreated) or AAV-CasRx/HTT gRNAs (Treated) at 3, 6, and 9 months of age and age-matched wild-type (WT) mice. Vinculin was used as a loading control. **B** Quantification of the ratios of NeuN, GFAP, and Iba1 to vinculin on the western blots. *n* = 4 mice per group. Data were analyzed by one-way ANOVA and presented as mean ± SEM. NeuN: WT vs Treated ( *P* = 0.9922 (3 M), *P* = 0.2382 (6 M), *P* = 0.8823 (9 M)), Treated vs Untreated ( *P* = 0.8593 (3 M), *P* = 0.9602 (6 M), *P* = 0.9698 (9 M)); GFAP: WT vs Treated ( *P* = 0.9366 (3 M), *P* = 0.0588 (6 M), ****P < 0.0001 (9 M)), Treated vs Untreated ( **P = 0.0042 (3 M), ***P = 0.0010 (6 M), ***P = 0.0002 (9 M)); Iba1: WT vs Treated ( *P* = 0.2684 (3 M), **P = 0.0013 (6 M), ****P < 0.0001 (9 M)), Treated vs Untreated ( ***P = 0.0004 (3 M), *P = 0.0258 (6 M), ****P < 0.0001 (9 M)). **C** Representative micrographs of immunofluorescence staining of the striatum from HD KI-140Q mice injected with AAV-CasRx/Ctrl gRNA (Untreated) or AAV-CasRx/HTT gRNAs (Treated) at 3, 6, and 9 months of age. Brain slices were examined after 1.5 months of viral injection and stained with antibodies to GFAP, Iba1 and GFP. GFP indicated gRNA expression. Scale bars: 50 μm. **D** Quantification of the numbers of GFAP-positive or Iba1-positive cells. Data are presented as mean ± SEM. *n* = 6 animals per group, two-way ANOVA were used for statistical analysis. GFAP, **P = 0.0055 (3 M); **P = 0.0073 (6 M); *P* = 0.0528 (9 M). Iba1, *P* = 0.1423 (3 M); **P = 0.0070 (6 M); *P = 0.0152 (9 M)

We investigated whether CRISPR/CasRx treatment could reverse the typical neuropathology of HD 140Q-KI mice, which includes increased gliosis with nuclear accumulation and aggregation of mHTT [56, 76, 77]. Western blotting using glial fibrillary acidic protein (GFAP) [80–82], a marker of astrocytes and ionized calcium binding adapter molecule 1 (Iba1) [83, 84], a marker of microglia, showed that the increase of GFAP and Iba1 were reverted 6 weeks after injecting AAV-CasRx/HTT gRNAs into the striatum of HD 140Q-KI mice at different ages (Fig. 3A, B). At the same time, immunofluorescence results were consistent with our previous study in pigs [37]. In WT mice, we did not observe significant proliferation of glial cells after AAV9 injection compared with injection of PBS (Extended Data Fig. 3A). In addition, consistent with the WB results mentioned above, administration of AAV-CasRx/HTT gRNAs effectively reduced the number of GFAP-positive and IBA1-positive cells (Fig. 3C, D). We also examined the number of neurons labeled with neuronal nuclear protein (NeuN) [85], and the results of western blotting and immunofluorescence staining consistently showed that CRISPR/CasRx treatment did not change the number of neurons (Fig. 3A, B; Extended Data Fig. 4A). This was due to the limitations of HD 140Q-KI mice, which do not have significant neuronal loss [77, 86–88]. Therefore, no significant changes in the number of neurons were observed in HD 140Q-KI mice after CRISPR/CasRx treatment. In summary, the above experimental results support the notion that CRISPR/CasRx treatment can reverse the increased gliosis in the striatum of HD 140Q-KI mice.

**Fig. 4:**
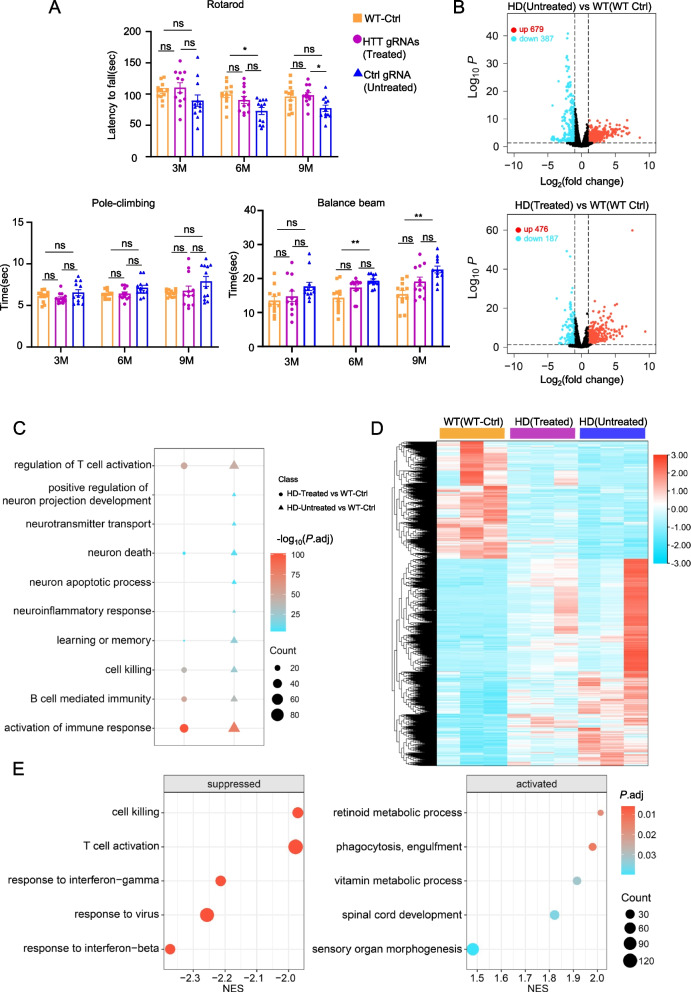
CRISPR/CasRx alleviated the motor dysplasia and mitigated gene expression dysregulation in the HD 140Q-KI mice. **A** Motor functions of WT mice injected AAV-Ctrl gRNA and HD KI-140Q mice injected with AAV-CasRx/Ctrl gRNA (Untreated) as control or with AAV-CasRx/HTT gRNAs (Treated). Mice were injected at 3, 6, and 9 months of age and examined 1.5 months after injection. *n* = 12 mice per group. Data were analyzed by two-way ANOVA and presented as mean ± SEM. Rotarod: WT vs Treated ( *P* = 0.8453 (3 M), *P* = 0.5126 (6 M), *P* = 0.9421 (9 M)), Treated vs Untreated ( *P* = 0.2108 (3 M), *P* = 0.1780 (6 M), *P = 0.0212 (9 M)), WT vs Untreated ( *P* = 0.2834 (3 M), *P = 0.0188 (6 M), *P* = 0.0719 (9 M)); Pole-climbing: WT vs Treated ( *P* = 0.5147 (3 M), *P* = 0.9058 (6 M), *P* = 0.8507 (9 M)), Treated vs Untreated ( *P* = 0.2330(3 M), *P* = 0.1471 (6 M), *P* = 0.3280 (9 M)), WT vs Untreated ( *P* = 0.5805 (3 M), *P* = 0.1068 (6 M), *P* = 0.0866 (9 M)); Balance beam: WT vs Treated ( *P* = 0.4292 (3 M), *P* = 0.1162 (6 M), *P* = 0.0529 (9 M)), Treated vs Untreated ( *P* = 0.3078 (3 M), *P* = 0.1553 (6 M), *P* = 0.0597 (9 M)), WT vs Untreated ( *P* = 0.0659 (3 M), **P = 0.0072 (6 M), **P = 0.0012 (9 M)). **B** Differential expressed genes (DEGs) while AAV-CasRx/HTT gRNAs (Treated)- or AAV-CasRx/Ctrl gRNA (Untreated)-injection HD-KI mice versus AAV-Ctrl gRNA (WT-Ctrl)-injection WT mice; volcano plot indicates Group HD (Untreated) versus Group WT (WT-Ctrl) with 1066 total DEGs (387 downregulated; 679 upregulated) while Group HD (Treated) versus Group WT (WT-Ctrl) with 663 DEGs(187 downregulated; 476 upregulated) that much less than untreated HD-KI mice (*n* = 3, *P* < 0.05, foldchange ≥ 2). **C** Gene Ontology (GO) pathway enrichment analysis of two groups DEGs from (B) reveals AAV-CasRx/HTT gRNAs treatment can affect the neuronal related pathway genes on HD-KI mice. (*P* < 0.05). **D** Heatmap of the expressing levels (FKPM) of total DEGs from Group HD (Untreated) vs Group WT (WT-Ctrl) in HD (Untreated)/HD (Treated)/WT (WT Ctrl) mice shows the transcriptome of HD (Treated) mice are more similar to WT control mice than HD(Untreated) mice (*n* = 3). **E** Gene Set Enrichment Analysis (GSEA) of the differential analysis of group HD (Treated) versus group HD (Untreated) mice shows AAV-CasRx/HTT gRNAs treatment suppressed such immune-related pathways and activated the pathways like phagocytosis and others. (*P*.adj < 0.05)

### CRISPR/CasRx treatment partially mitigated gene expression dysregulation in the HD 140Q-KI mice striatum

Previous studies have demonstrated that motor dysfunction in HD 140Q-KI mice can be assessed through rotarod and balance beam tests [56, 77]. Similarly, we performed behavioral tests on WT mice injected Ctrl gRNA and HD 140Q-KI mice injected with CasRx and HTT gRNAs or Ctrl gRNA. We found that HD 140Q-KI mice treated with AAV-CasRx/HTT gRNAs at 6 and 9 months of age had improved motor performance compared with mice treated with AAV-CasRx/Ctrl gRNA (Fig. 4A). This result indicated that CRISPR/CasRx could improve the motor function of HD KI-140Q mice. We then selected HD 140Q-KI mice that were treated at 6 months of age to examine whether the deregulation of gene expression was reversed by CRISPR/CasRx treatment. We isolated striatum RNA from wild-type (WT) mice injected AAV-Ctrl gRNA, HD 140Q-KI mice injected with AAV-CasRx/Ctrl gRNA, and AAV-CasRx/HTT gRNAs for RNA sequencing (RNA-seq) analysis. The volcano plot of differential analysis shows that the AAV-CasRx/HTT gRNAs treatment HD-KI mice have fewer differentially expressed genes (DEGs) (665 DEGs) compared to the untreated AAV-CasRx/Ctrl gRNA HD-KI mice relative to the WT mice injected Ctrl gRNA (1066 DEGs). (*P* < 0.05, foldchange ≥ 2) (Fig. 4B). In HD 140Q-KI mice injected with AAV-CasRx/Ctrl gRNA, DEG were enriched in HD-related pathological pathways, and knockdown of *HTT* mRNA by CRISPR/CasRx effectively alleviated some gene transcription dysregulation related to immune and inflammatory responses and neuronal projection (Fig. 4C). DEG enrichment analysis found that compared with HD 140Q-KI mice injected with AAV-CasRx/Ctrl gRNA, the gene expression levels of HD 140Q-KI mice treated with CRISPR/CasRx were closer to those of WT mice used as control (Fig. 4D). We conducted Gene Set Enrichment Analysis (GSEA) analysis on the differential analysis results of AAV-CasRx/HTT gRNAs treated HD-KI mice versus control HD-KI mice. The findings indicated that treatment with AAV-CasRx/HTT gRNAs significantly inhibited immune and pro-inflammation related pathways, while simultaneously activating pathways such as phagocytosis, vitamin metabolism and neuronal-related pathways (*P.adj* < 0.05) (Fig. 4E). In conclusion, targeting *HTT* mRNA by CRISPR/CasRx can partially alleviate gene expression dysregulation in HD 140Q-KI mice.

### Stereotactic injection of CRISPR/CasRx reduced mutant HTT expression in the striatum of HD-KI pigs

**Fig. 5:**
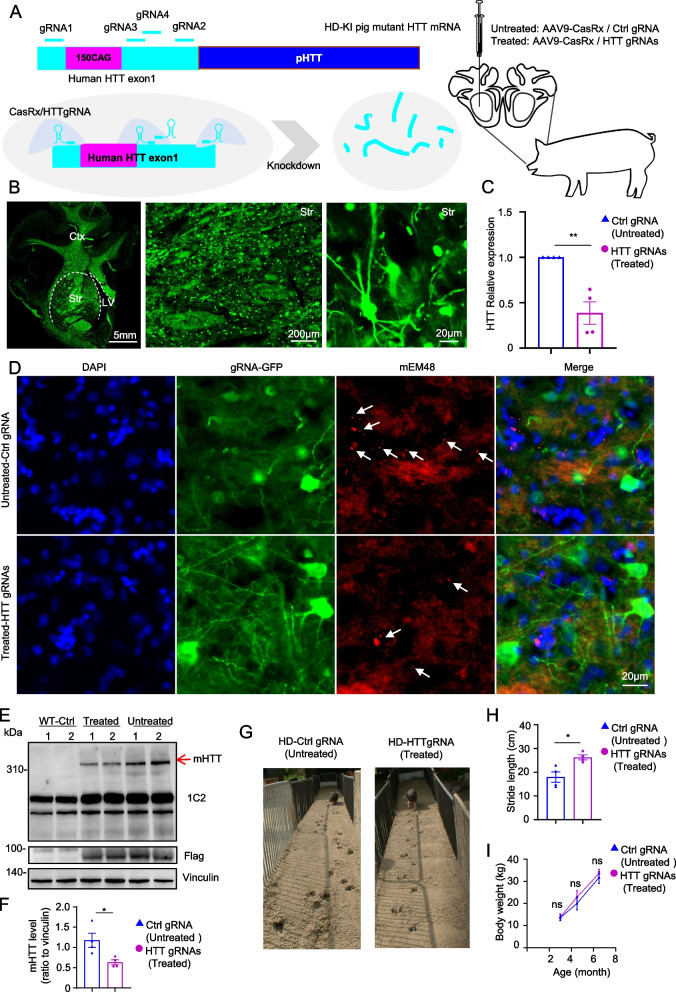
Analysis of HD-KI pig after stereotaxic injection with AAV-CasRx/HTT gRNAs. **A** Schematic diagram of CasRx-mediated knockdown of *HTT* mRNA in HD-KI pigs. AAV9 viruses carrying HTT gRNAs (gRNA1 and gRNA2; gRNA3 and gRNA4) and CasRx (Treated group) were delivered by brain stereotaxic injection into the striatum of HD KI pigs at 3 months of age. The control group (Untreated group) was injected with AAV9 viruses carrying CasRx and Ctrl gRNA. **B** Immunofluorescence staining with antibody against GFP showing expression of gRNA (gRNA constructs containing GFP tag) in a wide range of the pig’s striatum. ctx, cortex; Str, striatum; LV, lateral ventricle. On the right are the high magnification micrographs displaying GFP expression in the striatum. Scale bar: 5 mm (left), 200 μm (middle), 20 μm (right). **C** RT-qPCR analysis of *HTT* mRNA expression in HD-KI pigs treated at 3 months of age. *n* = 4 pigs per group. Data were analyzed by unpaired two-tailed t-test and presented as mean ± SEM. **P = 0.0026. **D** Double immunofluorescent images of brain sections (striatum) stained with antibodies GFP (gRNA) and mEM48 (mHTT). DAPI was used for nuclear staining. Scale bars: 20 μm. **E** Representative Western blots of mHTT (1C2) expression in HD-KI pigs treated at 3 months of age. Flag indicated the expression of CasRx (CAG-CasRx constructs containing flag tag) and vinculin was used as a loading control. **F **The quantification ratios of mHTT to vinculin. Unpaired two-tailed t-test was used for statistical analysis of two groups and data were expressed as mean ± SEM. Each group was four animals. *P = 0.0257. **G** The foot-printing test of HD-KI pigs injected with AAV-CasRx/Ctrl gRNA (left) and AAV-CasRx/HTT gRNAs (right). **H **Quantification of stride lengths for front and rear footprints of HD KI pigs, both untreated and treated with AAV-CasRx/HTT gRNAs injected into the striatum. The untreated pigs received injections of AAV-CasRx/Ctrl gRNA. Data are presented as mean ± SEM, with *n* = 4 animals per group. A statistically significant difference was observed (*P = 0.0140), determined using an unpaired two-tailed t-test. **I** Body weight of untreated and treated HD-KI pigs (*n* = 4 animals per group) was presented. Data are expressed as mean ± SEM, and with two-way ANOVA employed for statistical analysis

To extend our studies to large animal models of HD, we investigated the therapeutic effect of CRISPR/CasRx on heterozygous HD knock-in pigs carrying a mutant *HTT* allele with 150 CAG repeats [50]. Similar to HD 140Q-KI mice, HD-KI pigs carry endogenous *HTT* exon 1 that is replaced by human *HTT* exon 1 containing abnormally repetitive CAG repeats [50, 56]. Therefore, we wanted to treat HD-KI pigs with the four gRNAs targeting the mature human *HTT* exon1 mRNA (Fig. 5A). HD-KI pigs generally show HD-related pathology and movement disorders at the age of 3 months, and the striatum is the earliest and most severely affected brain region in HD-KI pigs [50]. Thus, we injected AAV-CasRx/gRNA into the bilateral striatum of 3-month-old HD-KI pigs by brain stereotaxic injection, and injected AAV-Ctrl gRNA into WT pigs of the corresponding age (Fig. 5A). Four months after virus injection, the pigs were euthanized after motor ability testing, and brain tissues were collected for the following analysis.

The results of immunofluorescence staining showed that GFP was widely expressed in the injected area, which was because the AAV9-gRNA carried the coding sequence of GFP (Fig. 5B). After confirming that the virus was successfully expressed, we evaluated the therapeutic effect of CRISPR/CasRx on HD-KI pigs at both the RNA and protein levels. Due to the high similarity between the *HTT* exon1 sequences of pigs and humans, specific primers capable of distinguishing mutant *HTT* mRNA from WT *HTT* mRNA in HD-KI pigs were not available. As a result, we could only identify changes in total *HTT* mRNA. The RT-qPCR results demonstrated a significant reduction of total *HTT* mRNA in the treatment group injected with AAV-CasRx/HTT gRNAs compared to HD-KI pigs injected with AAV-CasRx/Ctrl gRNA (Fig. 5C). In addition, we detected the expression of mHTT in the striatum of 7-month-old HD-KI pigs injected with AAV-CasRx/gRNA at 3 months of age through immunostaining and western blotting. Both aggregated mHTT (Fig. 5D; Extended Data Fig. 5A) and full-length mHTT (Fig. 5E) were significantly reduced after AAV-CasRx/HTT gRNAs injection compared with control striatum injected with control gRNA, which was also verified by quantitative analysis (Fig. 5F; Extended Data Fig. 5B).

In addition to the typical pathological features of the production of aggregated mHTT, HD patients exhibit typical involuntary choreiform movements accompanied by severe voluntary movement deficits, which were also observed in HD-KI pigs [50, 89, 90]. The motor performance of 7-month-old HD KI pigs injected with AAV-CasRx/gRNA was assessed by analyzing their performance over sandy tracks. HD-KI pigs injected with AAV-Ctrl gRNA (untreated) showed an irregular gait and shorter stride lengths, whereas HD-KI pigs injected with AAV-HTT gRNAs/CasRx exhibited much improved gait performance (Fig. 5G, H; Supplementary video-1). Nonetheless, the brain injection of AAV-CasRx/HTT gRNAs did not have a significant impact on the body weight of HD KI pigs (F ig. 5I). In our previous experiments, we employed treadmill performance as a measure to assess the locomotor capacity of pigs [37, 50]. In this study, we conducted treadmill experiments on the same HD-KI pig before and after treatment. Following treatment with CasRx, a significant improvement in the motor function of HD pigs was observed (Extended Data Fig. 6A; Supplementary video-2).

**Fig. 6:**
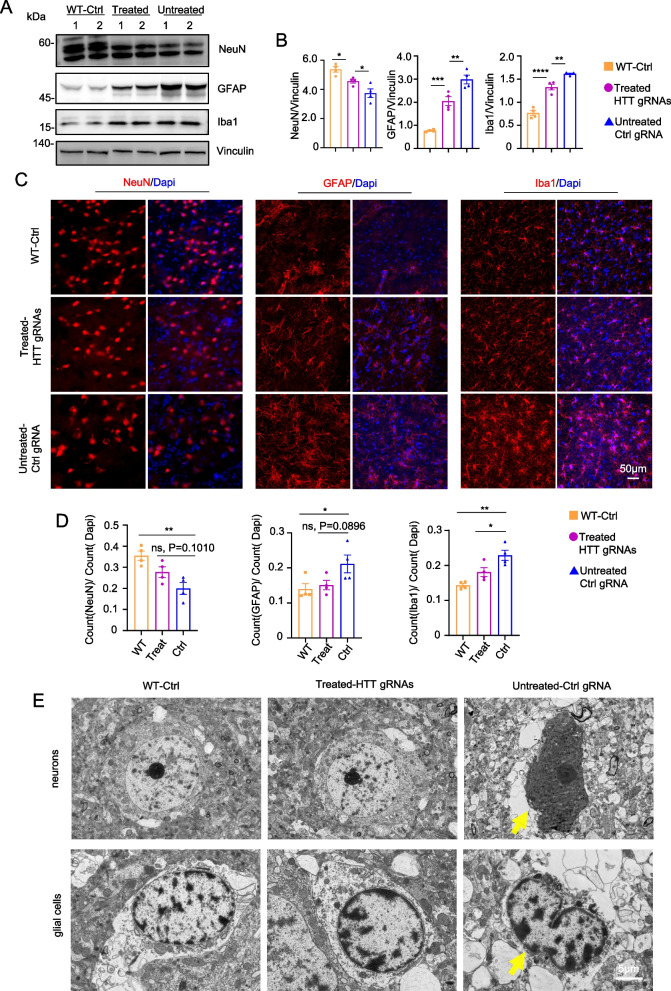
Analysis of the neuropathology in HD-KI pigs after AAV-CRISPR/CasRx treatment. **A** Western blot analysis of the injected pig brain striatum tissues with antibodies to NeuN, GFAP, and IBA1 in WT pigs injected AAV-Ctrl gRNA, AAV-CasRx/HTT gRNA (Treated)- or AAV-CasRx/Ctrl gRNA (Untreated)-injection HD-KI pigs. Vinculin was used as a loading control. **B** Quantification of the ratios of NeuN, GFAP, and IBA1 to vinculin on the Western blots. *n* = 4 pigs per group. Data were analyzed by one-way ANOVA and presented as mean ± SEM. NeuN: WT vs Treated (*P = 0.0457), Treated vs Untreated (*P = 0.0428); GFAP: WT vs Treated (***P = 0.0005), Treated vs Untreated (**P = 0.0040); Iba1: WT vs Treated (****P < 0.0001), Treated vs Untreated (**P = 0.0038). **C** Representative immunofluorescent fluorescent images of the striatum from WT pigs injected AAV-Ctrl gRNA, AAV-CasRx/HTT gRNAs (Treated)- or AAV-CasRx/Ctrl gRNA (Untreated)-injection HD-KI pigs. Antibodies for NeuN, GFAP, Iba1 were used. Scale bars: 50 μm. **D** Quantification of the numbers of NeuN-positive, GFAP-positive and Iba1-positive cells. *n* = 4 animals per group. Data were analyzed by one-way ANOVA and presented as mean ± SEM. NeuN: WT vs Untreated (**P = 0.0034), Treated vs Untreated (*P* = 0.1010); GFAP: WT vs Untreated (*P = 0.0451), Treated vs Untreated (*P* = 0.0896); Iba1: WT vs Untreated (**P = 0.0011), Treated vs Untreated (*P = 0.0321). **E** Representative micrographs of electron microscopy examination of the neuronal and reactive glial morphology in WT pigs injected AAV-Ctrl gRNA (WT Ctrl), and HD-KI pigs (Treated and Untreated). The untreated HD-KI pigs displayed degenerated neurons that appeared as dark neurons (arrows). The degenerated neurons show an electron-lucent cytoplasmic region containing degenerated organelles and irregular and disintegrated nuclear membranes. In the untreated HD-KI pig striatum, reactive glial cells exhibit electron-dense and tightly clustered heterochromatin, which is distinctly accumulated beneath the nuclear envelope, along with variable-sized cytoplasmic vacuoles (arrows). Scale bars: 5 μm

### Injection of CRISPR/CasRx partially alleviates neuropathology in the striatum of HD-KI pigs

Since HD-KI pigs display neurodegeneration, we then investigated whether brain injection of CRISPR/CasRx could alleviate this important pathological feature. We focused on neuropathology observed in both HD patients and HD-KI pigs, including neuronal loss and gliosis [50, 91]. Four months after the injection of AAV-CasRx/gRNA into the striatum of HD-KI pigs, we examined the changes in NeuN, GFAP, and IBA1 expression levels using western blotting. The results showed that the pathological changes of NeuN reduction, GFAP and IBA1 increase were reversed after CasRx/HTT gRNAs treatment (Fig. 6A, B). The results of immunofluorescence staining were consistent with those of western blotting. That is, the knockdown of *HTT* mRNA by CRISPR/CasRx significantly reduced neuronal death and effectively alleviated the proliferation of astrocytes and microglia (Fig. 6C, D). Many degenerated neurons were found in the striatum of HD-KI pigs [50], but the results of electron microscopy showed most of the neurons exhibited normal morphology similar to the wild type neurons after treatment with CRISPR/CasRx. We also observed glial cells with highly condensed heterochromatin in the untreated group as our previous research (Fig. 6E) [50]. These findings suggest that CRISPR/CasRx-mediated knockdown of *HTT* mRNA has therapeutic effects on attenuating neurodegeneration and reducing gliosis in HD-KI pig brains.

### CRISPR/CasRx treatment partially mitigated gene expression dysregulation in the striatum of HD KI pigs

To evaluate the potential off-target effects of AAV-CasRx/HTT gRNAs, we screened 5 candidate off-target transcripts for each HTT gRNA by aligning on the whole genome of pigs, and a total of 20 candidate off-target genes were selected. Each candidate off-target gene has at least 10 consecutive nucleotides consistent with the corresponding HTT gRNAs (Extended Data Fig. 7A). Out of these 20 candidate genes, one gene was not detected in any of the experimental groups. For the other 19 genes, we found no significant expression differences in HD-KI pigs injected with AAV-CasRx/Ctrl gRNA or AAV-CasRx/HTT gRNAs, when compared to the WT control injection group (Extended Data Fig. 7B). In addition, RT-qPCR analysis revealed no significant differences in the expression of all candidate genes in HD-KI pigs treated with CasRx/HTT gRNAs compared to those injected with AAV-CasRx/Ctrl gRNA (Extended Data Fig. 7C). These findings demonstrate the high specificity of CRISPR/CasRx-mediated RNA knockdown in the treatment of HD-KI pigs.

**Fig. 7:**
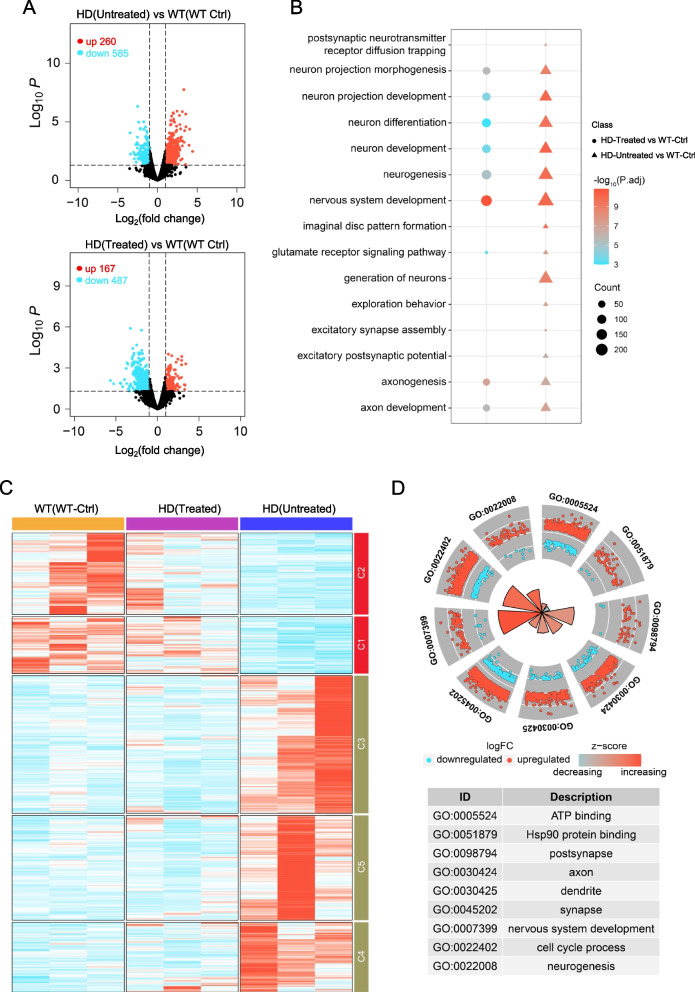
RNA-Seq Analysis reveals the neuropathological alleviation in HD-KI pigs after AAV-CRISPR/CasRx treatment. **A** DEGs while AAV-CasRx/HTT gRNAs (Treated)- or AAV-CasRx/Ctrl gRNA (Untreated)-injection HD-KI pigs versus WT pigs injected AAV-Ctrl gRNA (WT Ctrl); volcano plot indicates Group WT (WT Ctrl) versus HD (Untreated) with 845 total DEGs (585 downregulated; 260 upregulated) as well as WT (WT Ctrl) versus Group HD (Treated) with 654 DEGs (487 downregulated; 167 upregulated) that lower than untreated HD-KI pigs (*n* = 3, *P* < 0.05, foldchange ≥ 2). **B** Gene Ontology (GO) pathway enrichment analysis of two groups DEGs from (A) reveals AAV-CasRx/HTT gRNAs treatment alleviates the neuronal impairment in HD-KI pigs. (*P* < 0.05). **C** Heatmap of the expressing levels (RPM-normalized) of total DEGs from Group HD (Untreated) vs WT (WT Ctrl) in HD(Untreated)/HD(Treated)/WT(WT Ctrl) pigs shows the transcript dysfunctions of HD-KI pigs were recovered after AAV-CasRx/HTT gRNAs treatment (*n* = 3, Cluster K-means = 5). **D** Gene Ontology (GO) pathway enrichment of the total DEGs from Group HD(Untreated) versus HD(Treated) shown by circle plot reveals that mostly neuron/axon/synase related genes dysregulated in HD pigs got rescued after AAV-CasRx/HTT gRNA treatment. (*P.adj* < 0.05)

Due to the impact of mHTT on the transcription process [11, 12], its accumulation in the striatum can lead to dysregulation of gene expression in both HD patients and several HD animal models [37, 92–95]. In order to investigate whether the dysregulation of gene expression in the striatum of HD-KI pigs could be reversed by knocking down *HTT* mRNA using CRISPR/CasRx, we conducted gene expression analysis using striatum from WT pigs injected AAV-Ctrl gRNA and HD-KI pigs injected with AAV-CasRx/Ctrl gRNA or AAV-CasRx/HTT gRNAs. RNA was isolated from the striatum for subsequent RNA-Seq analysis. The volcano plot of differential analysis shows that the AAV-CasRx/HTT gRNAs treatment HD-KI pigs have fewer differentially expressed genes (654 DEGs) compared to the untreated AAV-CasRx/Ctrl gRNA HD-KI pigs relative to the WT injection control group (845 DEGs). (*P* < 0.05, foldchange ≥ 2) (Fig. 7A). Importantly, the reversed DEGs were found to be enriched in each of the HD-associated pathological pathways, including neuronal projection, axon, neuronal development, and synaptic signaling (Fig. 7B).This suggests that our gene therapy approach has the potential to alleviate the downstream effects of mHTT that have been reported in previous studies [96–98]. We conducted an enrichment analysis on the DEGs within each group (Extended Data Fig. 8A). Our findings revealed significant disparities between the WT pigs injected AAV-Ctrl gRNA and HD-KI pigs that were injected with AAV-CasRx/Ctrl gRNA. However, these differences were notably diminished following injection of AAV-CasRx/HTT gRNAs (Fig. 7C). We also performed differential analysis between AAV-CasRx/HTT gRNAs injected HD-KI pigs and AAV-CasRx/Ctrl gRNA injected HD-KI pigs and then conducted GO enrichment analysis on the differentially expressed genes (*P* < 0.05, foldchange ≥ 2). The enrichment results showed that the relative DEGs after AAV-CasRx/HTT gRNAs treatment were predominantly enriched in pathways related to neuron/axon/synapse (*P.adj* < 0.05) (Fig. 7D).

## Discussion

In this study, we used the CRISPR/CasRx system to effectively down-regulate *HTT* mRNA and subsequently reduce the expression of HTT in 293 T cells. In addition, we co-delivered AAV9 vectors carrying CasRx and HTT gRNAs to the striatum of HD 140Q-KI mice at different stages of the disease and HD-KI pigs, resulting in a significant reduction in the expression of mHTT. Notably, this approach also led to improvements in gliosis in HD 140Q-KI mice [56], as well as mitigated neurodegeneration and dysregulation of gene expression in HD-KI pigs [50]. These findings strongly indicate that CRISPR/CasRx-mediated RNA editing holds promise as a novel therapeutic strategy for HD, as it effectively reduces mHTT expression by targeting *HTT* mRNA and alleviates HD-associated neuropathology.

HD is a neurodegenerative disease caused by a single-gene mutation, and mHTT plays a key role in the development of the disease [1, 2, 4]. The mutant protein has a significant impact on various physiological processes such as transcription [11, 12], synaptic function [99], axonal transport [7, 10], and mitochondrial function [100, 101]. Thus, developing drugs that target these affected downstream pathways is complex and challenging. So far, there are no successful therapeutic strategies specifically designed for these downstream pathways [102], suggesting that focusing on drug screening and development for the injured downstream pathways may not be the most effective approach. Instead, direct inhibition of mHTT expression is considered to be the most promising strategy for HD treatment [22]. According to the central dogma of molecular biology [103], blocking the expression of extended polyQ proteins can be achieved by targeting either the *HTT* DNA or mRNA. At present, CRISPR/Cas9 has been used in various HD models to knockout the *HTT* DNA for the treatment [34–37]. Typically, CAS9 is delivered by AAV vector, but the capacity of AAV vectors to accommodate foreign DNA is limited [104, 105], and the large size of the DNA sequence encoding the Cas9 protein [106] can negatively impact the packaging efficiency of the virus.

Consequently, the viral titers may be insufficient, posing limitations for future clinical applications. Additionally, since CRISPR/Cas9 permanently alters the sequence of the genome [38], the potential risks of off-target effects must be considered. In addition to targeting *HTT*, researchers have also explored approaches to enhance the clearance of mHTT through the intracellular antibody (intrabody) targeting HTT [107–112]. This approach shows promise for HD treatment as well. However, recent studies have revealed that in addition to mutated polyQ proteins, RNA transcripts carrying abnormal expanded CAG repeats are also toxic [113, 114]. These mutated RNA transcripts form abnormal CAG repeat hairpin motifs, which interfere with normal cellular functions by recruiting RNA-binding proteins abnormally [115, 116]. Therefore, it is necessary to prevent the accumulation of the toxic RNA carrying an abnormal number of CAG repeats in advance to prevent the accumulation of expanded polyQ proteins, in order to achieve better therapeutic effect.

At present, strategies for reducing mHTT expression by targeting *HTT* RNA primarily involve the use of ASO and RNAi. Among these strategies, intrathecal delivery of ASO has been shown to effectively reduce the expression of mHTT in the cerebrospinal fluid of HD patients [22]. Targeting *HTT* mRNA with microRNA reduced mHTT in HD transgenic pigs [26], and these results demonstrate the potential of inhibiting HTT mRNA expression as a therapeutic approach for HD. With the advancement of gene editing technology, the CRISPR/Cas13 RNA editing tool has been applied to the research and treatment of a variety of diseases, including genetic deafness [117], ALS [55], Duchenne muscular dystrophy (DMD) [118], and HD [44, 55], due to its high knockdown efficiency and low off-target effects. In addition, it has great potential in the treatment of diseases caused by RNA viruses. It can directly degrade viral RNA and inhibit viral replication. However, further investigation is needed to determine whether CRISPR/CasRx can be used to knock down *HTT* mRNA in large animals to alleviate neurodegeneration. Since rodent models of HD lack striking neurodegeneration, addressing this issue using HD-KI pigs is important and will contribute to the success of clinical trials of therapies using CRISPR/CasRx to knock down *HTT* mRNA.

In our study, our results demonstrated that CRISPR/CasRx-mediated RNA editing can rescue HD animal models, including the HD 140Q-KI mouse model [56] at different disease stages and the HD-KI pig model [50]. Although CRISPR/CasRx has therapeutic effects on HD 140Q-KI mice at different disease stages, the effects appear to be dependent on the disease stages. Treatment administered at earlier stages of the disease showed more significant benefits compared to treatment initiated at later stages. This finding is consistent with our understanding of the disease progression, as the proliferation of glial cells and accumulation of aggregates intensify as the disease advances [56], thereby reducing the efficacy of treatment in the middle and late stages. Consequently, it is crucial to identify the optimal timing for therapeutic intervention, with early treatment generally yielding better outcomes. Furthermore, we successfully delivered AAV9-CasRx/HTT gRNAs into the striatum of HD-KI pigs and observed a reduction in mHTT accumulation, as well as improvements in gene expression dysregulation and motor deficits. The more exciting finding is that CRISPR/CasRx-mediated RNA targeting, like mHTT DNA targeting, can also effectively reduce neurodegeneration, further supporting the potential of CRISPR/CasRx as a novel therapeutic strategy for HD. In this study, the inclusion of a limited number of HD KI pigs was noted. However, our analysis revealed a consistent treatment effect, with HD-KI pigs in the treatment group demonstrating improvements in HD-related pathology. Nonetheless, for future applications of CRISPR/CasRx targeting HTT RNA in HD patient treatment, a larger sample size of animals in the experiment is imperative to ensure robustness and reliability of the findings.

Because gRNA has the potential to target non-target genes and Cas13 family of enzymes has promiscuous RNase activity, CasRx may nonspecifically cut surrounding RNA (bystander RNA) [119–122]. Therefore, it is important to test the specificity of CRISPR/CasRx in targeting *HTT* mRNA. In our study, both RT-qPCR and RNA-seq results showed that the expression of 20 candidate off-target transcripts did not change significantly after knocking down *HTT* mRNA using CRISPR/CasRx in HD-KI pigs. Previously, some researchers have used CRISPR/CasRx to inhibit the expression of *HTT* [44, 55], *SOD1* [55], *ATXN2* [43]*, C9ORF72* containing the abnormal repeat sequence of GGGGCC [42], and *Tmc1* [117] in the mouse central nervous system, and no obvious off-target events were found. However, cas13 itself still has promiscuous RNase activity, and studies have shown that this incidental activity is positively correlated with the abundance of the target RNA [123]. Therefore, the specificity and safety of using CRISPR/CasRx for RNA knockdown are still important issues that need further research.

## Conclusion

Overall, we successfully rescued HD 140Q-KI mice at different disease stages by knocking down *HTT* mRNA using CRISPR/CasRx. Additionally, we validated the effectiveness of CRISPR/CasRx in treating HD by employing the HD-KI pig and demonstrated that this treatment can also mitigate the decline in motor function. These findings highlight the promising potential of CRISPR/CasRx for future therapeutic interventions not only in HD but also in other genetic mutation-related diseases.

## Supplementary Information

Supplementary Material 1.

## Data Availability

All data presented in this study are accessible within the main text or the supplementary materials. All raw sequencing data generated in this study have been deposited at NCBI’s Gene Expression Omnibus repository and are accessible under accession numbers GSE248873. For any additional data pertaining to this paper may contact the authors for further information or requests.

## Abbreviations

HD: Huntington's disease
*HTT*: Huntingtin
AD: Alzheimer's disease
PD: Parkinson's disease
ALS: Amyotrophic lateral sclerosis
ASO: Antisense oligonucleotide
mHTT: Mutant huntingtin
gRNA: Guide RNA
CasRx: Ruminococcus flavefaciens strain XPD3002
AAV: Adeno-associated virus
PAM: Protospacer adjacent motif
HD-KI pig: Huntingtin knock-in pig
HEK 293 T cells: Human embryonic kidney 293 T cells
RT-qPCR: Quantitative reverse transcription polymerase chain reaction
cDNA: Complementary DNA
GAPDH: Glyceraldehyde-3-phosphate dehydrogenase
EM: Electron microscopy
DEGs: Differentially expressed genes
GFAP: Glial fibrillary acidic protein
Iba1: Ionized calcium binding adapter molecule 1
NeuN: Neuronal nuclear protein
WT: Wild-type
RNA-seq: RNA sequencing
GSEA: Gene Set Enrichment Analysis
Intrabody: Intracellular antibody
DMD: Duchenne muscular dystrophy
PFS: Protospacer flanking sequence
