# Supplementary material for: RNA-Targeting CRISPR/CasRx system relieves disease symptoms in Huntington’s disease models
Source: Mol Neurodegener. 2025 Jan 13;20:4. doi: 10.1186/s13024-024-00794-w (PMC11727607; doi:10.1186/s13024-024-00794-w)
Supplement: Supplementary file 1 — Supplementary Material 1. [file 13024_2024_794_MOESM1_ESM.docx]

Supplementary material

Supplementary Figures


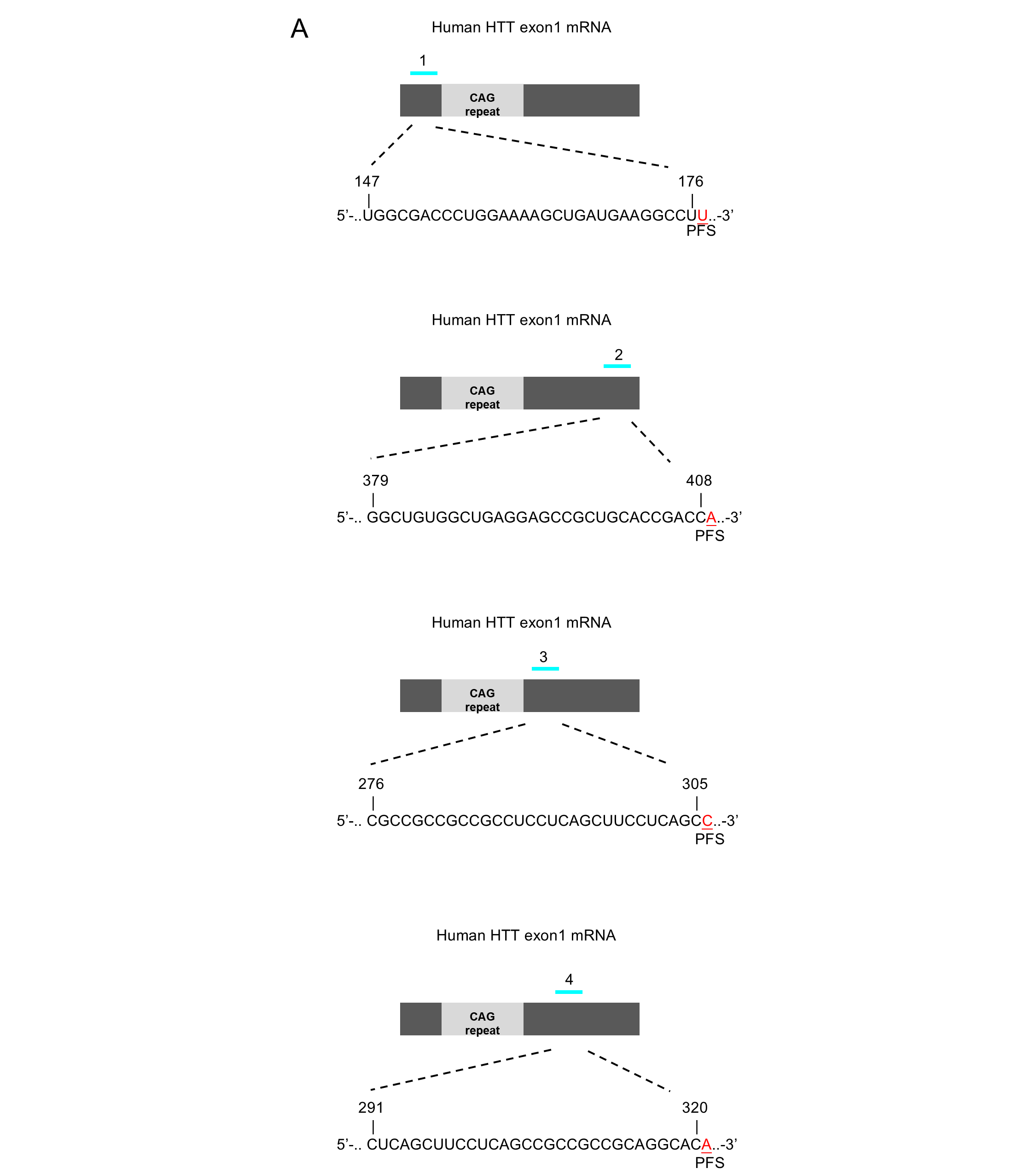


Extended Data Fig. 1 | Schematic of the human HTT exon1 mRNA. (A) Details about the protospacer flanking sequence (PFS) and recognition sites of each HTT gRNA.


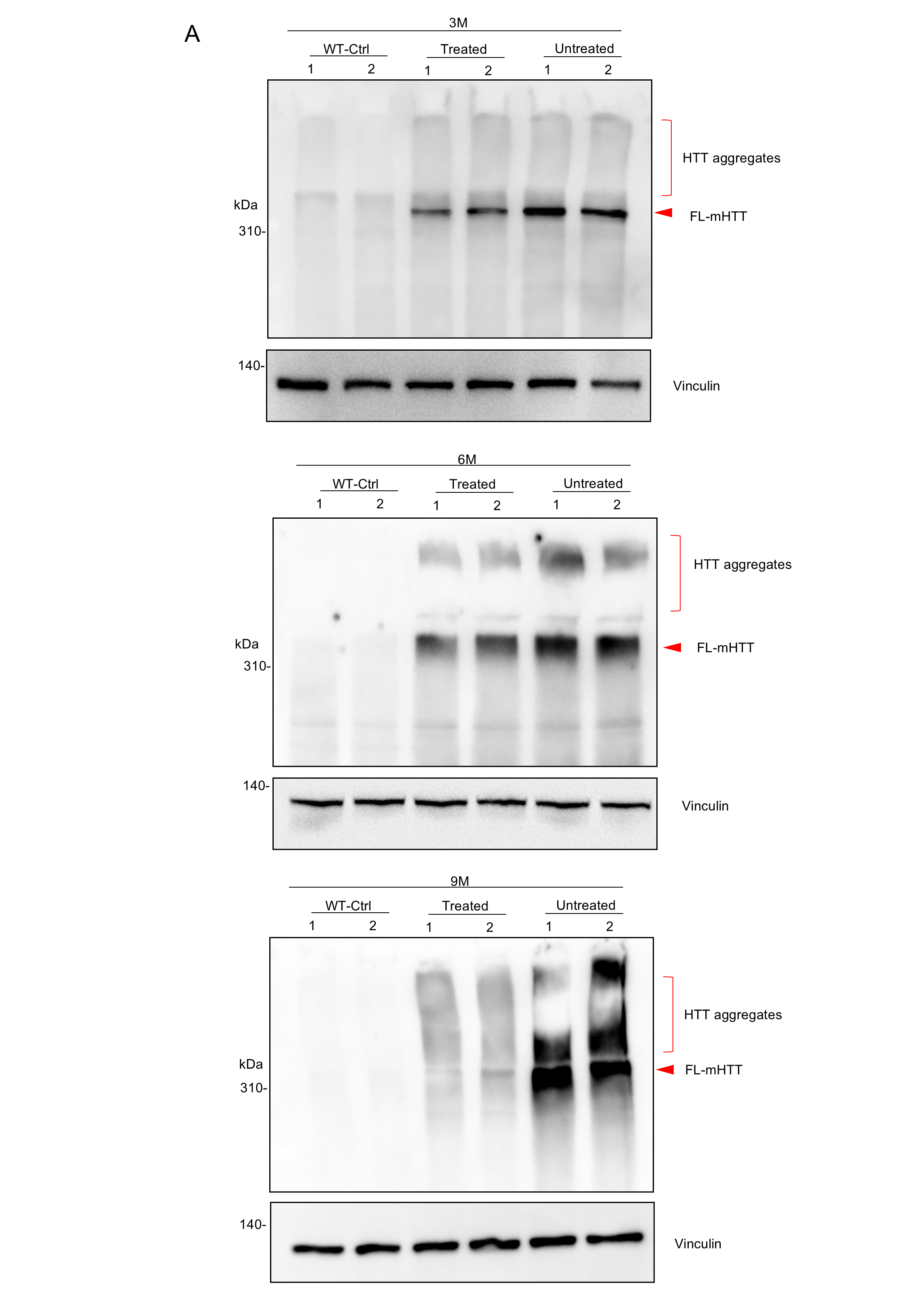
`

Extended Data Fig. 2 | CRISPR/CasRx reduced the expression of mutant HTT in the striatum of HD KI-140Q mice with different degrees of disease. (A) Representative Western blots of the striatum in HD KI-140Q mice were obtained one month after stereotaxic injection with AAV-CasRx/HTT gRNAs (Treated) or AAV-CasRx/Ctrl gRNA (Untreated), with treatments administered at 3, 6, and 9 months of age. The mEM48 antibody was used to detect the presence of mHTT and its aggregates, and vinculin was used as a loading control.


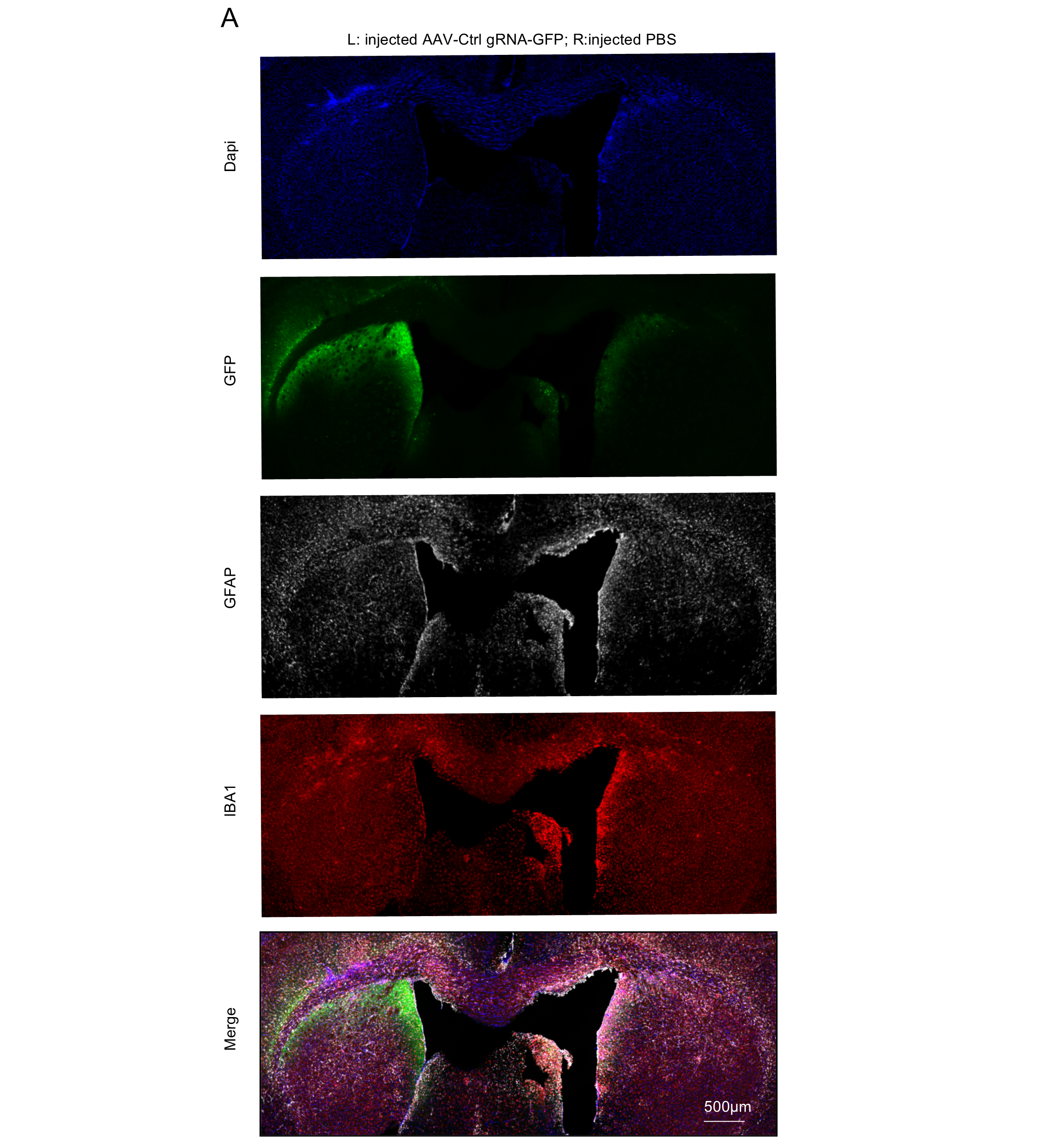


Extended Data Fig. 3 | Immunofluorescence staining of astrocytes and microglia of WT mice. (A) Immunofluorescence images of brain sections from mice injected with AAV-Ctrl gRNA-GFP on one side and PBS on the other side. Antibodies for GFAP and Iba1 were used. Scale bars: 500 μm.


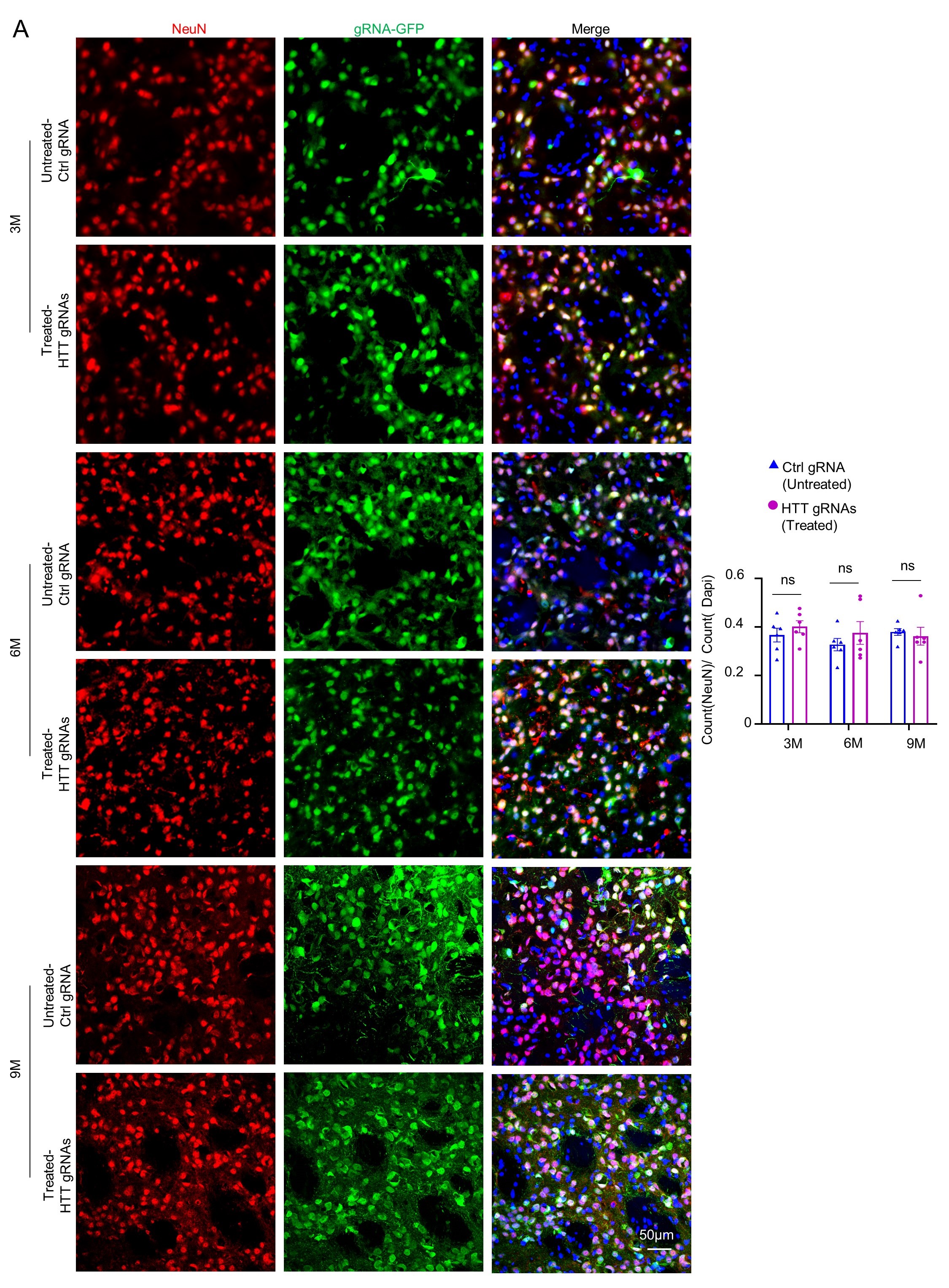


Extended Data Fig. 4 | Immunofluorescence staining of neurons in the striatum of HD KI-140Q mice with different degrees of disease. (A) Representative micrographs of immunofluorescence staining of the striatum were obtained from HD KI-140Q mice that were injected with AAV-CasRx/Ctrl gRNA (Untreated) or AAV-CasRx/HTT gRNAs (Treated) at 3, 6, and 9 months of age. Brain slices were examined 1.5 months after viral injection and stained with antibodies specific to NeuN and GFP. Scale bars: 50 μm. Quantification of NeuN-positive cells is presented below the staining. Data are expressed as mean ± SEM, with a sample size of 6 animals per group. Statistical analysis was performed using two-way ANOVA. The p-values for NeuN were as follows: *P* = 0.7582 (3M); *P* = 0.7822 (6M); *P* = 0.9649 (9M).


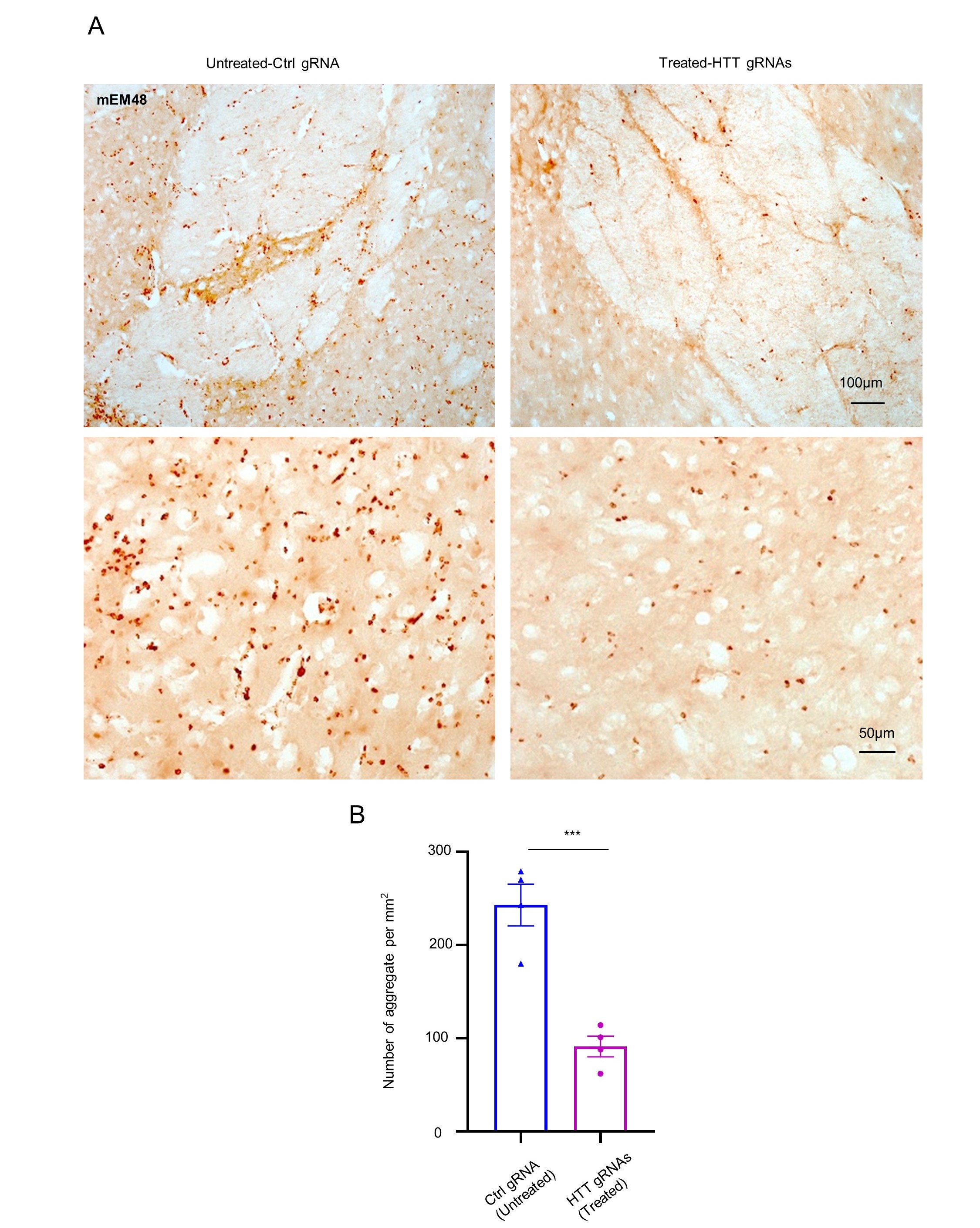


**Extended Data Fig. 5 | Immunohistochemistry staining of HTT aggregates in the striatum of HD-KI pigs.** (A) Representative immunohistochemistry micrographs of the striatum in HD-KI pigs injected with AAV-CasRx/Ctrl gRNA (Untreated), and AAV-CasRx/HTT gRNAs (Treated), stained with anti-HTT antibody (mEM48). Scale bar: 100 μm (top), 50 μm (below). (B) Quantification of the relative density of mHTT-positive cells per 0.1 mm^2^, with 4 animals per group. Statistical analysis was performed using an unpaired two-tailed t-test, and the data are presented as mean ± SEM. ****P* < 0.001.


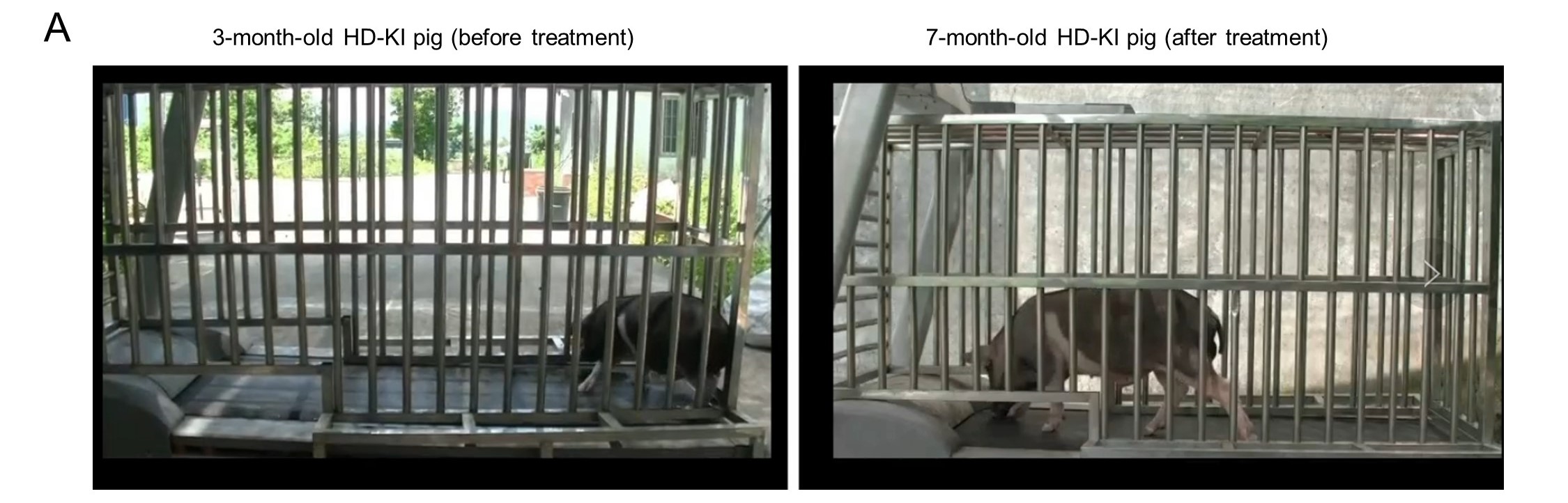


**Extended Data Fig. 6 | Treadmill analysis of the motor ability in HD-KI pig.** (A) Treadmill experiments on the same HD-KI pig both before (left) and after treatment (right).


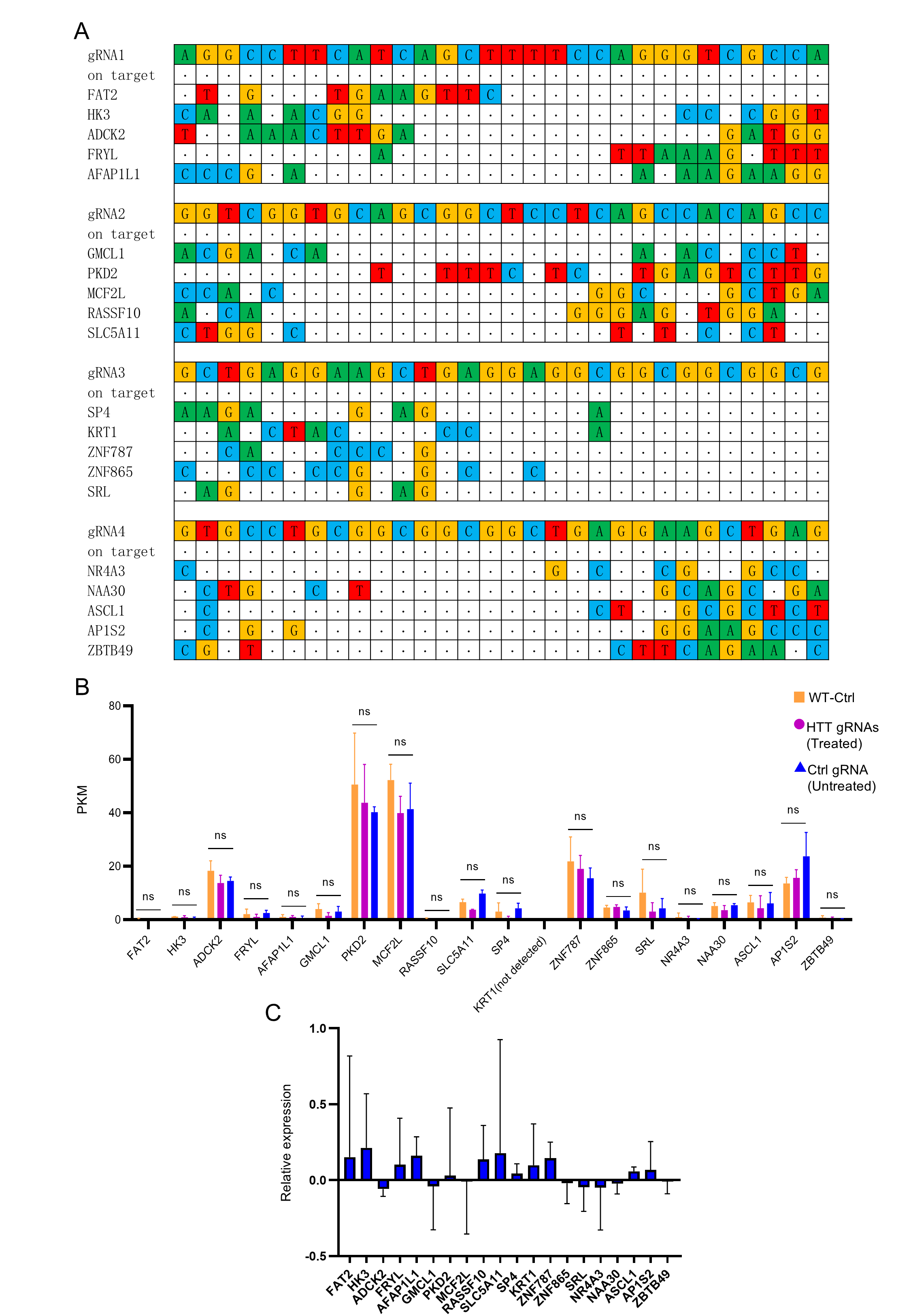


**Extended Data Fig. 7 | Off-target analysis of CRISPR/CasRx system for RNA editing in vivo.** (A) The target sequence of the HTT gRNAs and the candidate off-target sites with at least 10 contiguous nucleotides of homology are presented. Mismatches compared to the on-target site are highlighted in color. The 30 bp sequence (On-target) targeted by the HTT gRNAs were shown in the top row, respectively. (B) Off-target analysis in pigs was conducted using RNA-Seq. No significant difference was observed in the Treated group (injected with AAV-CasRx/HTT gRNAs) and the Untreated group (injected with AAV-CasRx/Ctrl gRNA) when compared to the WT group (injected with AAV-CAG-EGFP), expressing levels are normalized by reads per million (RPM). The data were presented as the mean ± SEM. "ns" indicates no significance. Statistical analysis was performed using multiple unpaired t-tests. (C) The normalized log2 fold-change of candidate off-target transcripts from HD-KI pigs injected with AAV-CasRx/HTT gRNAs relative to HD-KI pigs injected with AAV-CasRx/Ctrl gRNA (n=3) was shown. The fold-change was determined using the 2^-ΔΔCT^ method. The data were presented as the mean ± SEM. Statistical analysis was performed using one-way ANOVA. *P* > 0.05; not significant.


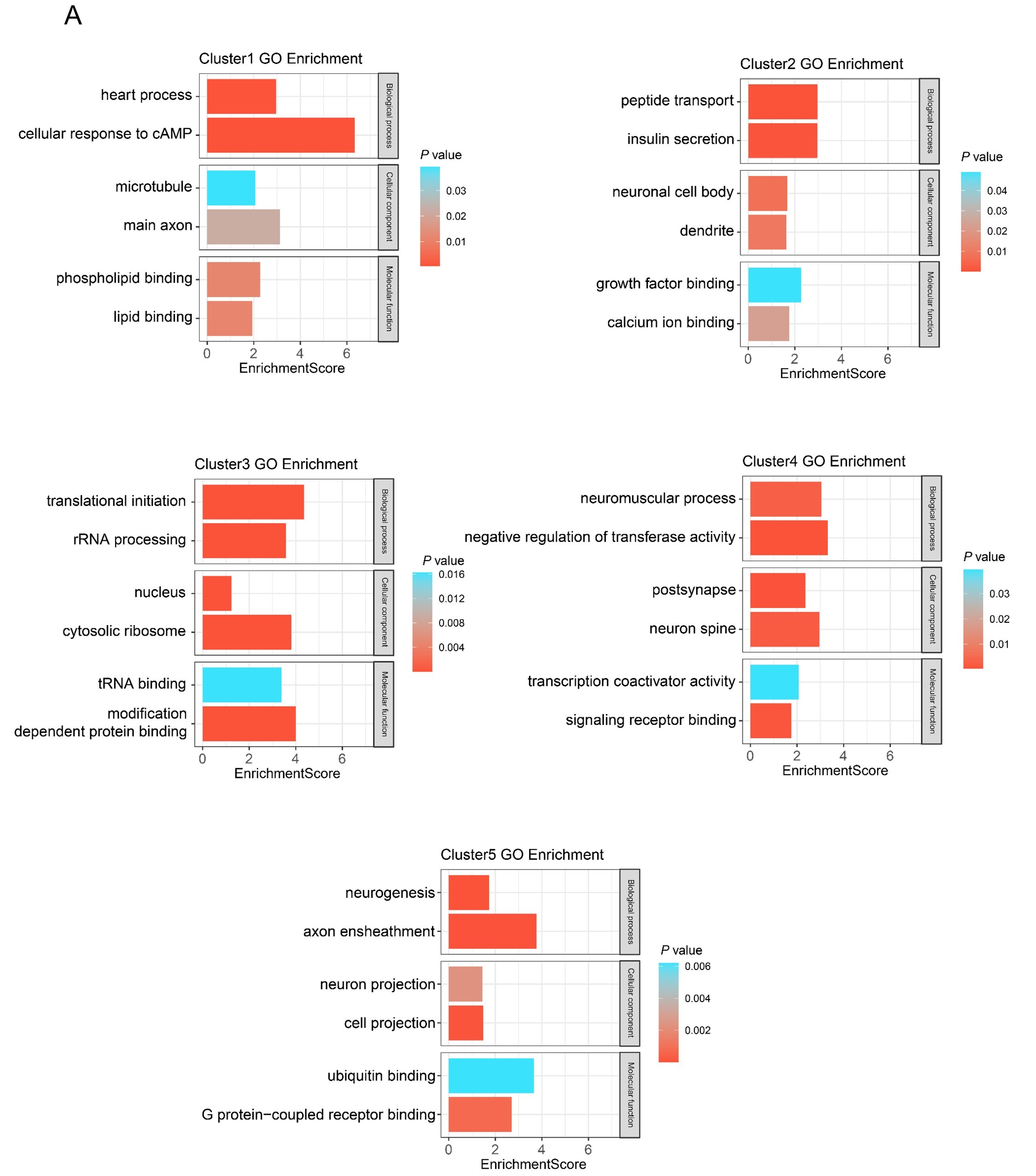


Extended Data Fig. 8 | Enrichment analysis of genes with the same expression trend in pig differential RNA-seq results. (A) GO enrichment analysis of clustering results in figure 7C were shown by barplot (*P* < 0.05).

Supplementary Videos

Supplementary Video 1: Foot print in sands of HD-KI brain injection of AAV-CasRx/Ctrl gRNA-untreated pigs and brain injection of AAV-CasRx/HTT gRNAs-treated pigs.


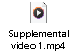


Supplementary Video 2: Treadmill performance of a HD-KI pig before (at 3 months of age) and after (at 7 months of age) treatment with CRISPR/CasRx.


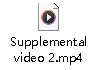


Supplementary Tables

Supplementary Table 1. Sequences of the oligonucleotides used in this study.

| Name | Sequence | |
| --- | --- | --- |
| Gene | Forward (5′ - 3′) | Reverse (5′ - 3′) |
| HTT gRNA1 | AGGCCTTCATCAGCTTTTCCAGGGTCGCCA |  |
| HTT gRNA2 | GGTCGGTGCAGCGGCTCCTCAGCCACAGCC |  |
| HTT gRNA3 | GCTGAGGAAGCTGAGGAGGCGGCGGCGGCG |  |
| HTT gRNA4 | GTGCCTGCGGCGGCGGCTGAGGAAGCTGAG |  |
| Ctrl gRNA | CGGAATTCATCCAGCCACCAGGGTCGCCG |  |
| qPCR-human-HTT | ATGGCGACCCTGGAAAAGCTGAT | TGAGGCAGCAGCGGCTG |
| qPCR-human-GAPDH | GCGAGATCCCTCCAAAATCAA | GTTCACACCCATGACGAACAT |
| qPCR-mouse-hHTT | GCCCGGCTGTGGCTGA | TTCACACGGTCTTTCTTGGTGG |
| qPCR-mouse-GAPDH | CTGAGCAAGAGAGGCCCTATCC | CTCCCTAGGCCCCTCCTGTT |
| qPCR-pig-HTT | AGGAGCCGCTGCACCGA | CTGAGAGACTGTGCCACTATGTT |
| qPCR-pig- GAPDH | ACCTGCCGCCTGGAGAAACC | GACCATGAGGTCCACCACCCTG |
| Genotyping-mouse | ACTGCTAAGTGGCGCCGCGTAG | GAGGCAGCAGCGGCTGTGCCTG |
| Genotyping-pig | GGAGAGCTGGGAGAGAATGCCAGTGTGACAGT | GCGGCTGAGGCAGCAGCGGCTGTGCCTG |
| qPCR-FAT2 | AGCAGCTCAGGTGTTGATGT | AATTCACTGCTCCGGGCAT |
| qPCR-HK3 | GCTGCTGTGGTGGAGAAGAT | AAGGTGACCTCACAGCAAGG |
| qPCR-ADCK2 | ACGAGTGCAAGGATGTGGAG | TTGAGGCGAAGTTGCTCTCC |
| qPCR-FRYL | AACTGCTGATCCACTTGCCT | AAGCCTGACAACTGGGAAGG |
| qPCR-AFAP1L1 | GGCAAAGGGAAGAAGAACAGC | AGCACGTTCAGGTGACCAC |
| qPCR-GMCL1 | ACTCCGAGACAGACGAGGAT | TTCCTTCGAGGGGTGTTGAG |
| qPCR-PKD2 | AGGTCTCTGGGGAACAAGACT | GTGGCCAGTTCCCGTAAAAC |
| qPCR-MCF2L | GAGCGTCCTGGAGAGCATC | TCGGTTTCATTCAGCTGGGC |
| qPCR-RASSF10 | ACGCTGGTGCATTTGGTACT | AACGTAGTTGACTCCGTGCC |
| qPCR-SLC5A11 | TGTTTCCCCAGAGGACGTTG | TTGCTGGCAAACAAGGATGC |
| qPCR-SP4 | AGTGTCTCCGTCTGAGGGTT | CTGGGAGCCTGAGGTTTTGG |
| qPCR-KRT1 | AGCAGGATGTCTGGAGAGTG | AACTTAATGCTGGAACTGCCAC |
| qPCR-ZNF787 | TGCGGAGACACAAGAAGGTC | GTAGTAGCTCTGTCCGCAGC |
| qPCR-ZNF865 | TCAAGAAACCCAGCCACCTC | TGCACACAGAGCAGGAGAAG |
| qPCR-SRL | CCTGGAGAAGCTGATTGGCA | CACACGTCGTTGAAGGGGTA |
| qPCR-NR4A3 | CCAGTTATGCAGCCCAGACA | CCAAGGTCCATGGTCAGCTT |
| qPCR-NAA30 | GACCAAGGTTTTGAGTGCGG | CGGCTCCTTTTGTTGCAGTC |
| qPCR-ASCL1 | ATGAGCATGACGCGGTGAG | TCCGACGAGTAGGATGAGACC |
| qPCR-AP1S2 | TGGATGAGTTTCTTTTGGGCG | AAATATTCATGACGTGGCTCCTGC |
| qPCR-ZBTB49 | GAAGGCCGTTCACCTGAAGA | CTGTGGACTTTCTCCACGCT |

Supplementary Table 2. Off-target sites.

The 20 off-target genes screened by aligning with sequence of HTT-sgRNAs in pigs.

| Gene name | Gene_chr | Gene_description |
| --- | --- | --- |
| FAT2 | 16 | FAT atypical cadherin 2 |
| HK3 | 2 | hexokinase 3 |
| ADCK2 | 18 | aarF domain containing kinase 2 |
| FRYL | 8 | FRY like transcription coactivator |
| AFAP1L1 | 2 | actin filament associated protein 1 like 1 |
| GMCL1 | 3 | germ cell-less 1, spermatogenesis associated |
| PKD2 | 8 | polycystin 2, transient receptor potential cation channel |
| MCF2L | 11 | MCF.2 cell line derived transforming sequence like |
| RASSF10 | 2 | Ras association domain family member 10 |
| SLC5A11 | 15 | solute carrier family 5 member 11 |
| SP4 | 9 | Sp4 transcription factor |
| KRT1 | 5 | keratin 1 |
| ZNF787 | 6 | zinc finger protein 787 |
| ZNF865 | 6 | zinc finger protein 865 |
| SRL | 3 | sarcalumenin |
| NR4A3 | 1 | nuclear receptor subfamily 4 group A member 3 |
| NAA30 | 1 | N-alpha-acetyltransferase 30, NatC catalytic subunit |
| ASCL1 | 5 | achaete-scute family bHLH transcription factor 1 |
| AP1S2 | X | adaptor related protein complex 1 subunit sigma 2 |
| ZBTB49 | 8 | zinc finger and BTB domain containing 49 |

Supplementary Table 3. Antibodies.

| Target | Host | Reference | Concentration |
| --- | --- | --- | --- |
| Anti-Rat IgG Alexa Fluor 594 | Goat | Invitrogen A-11007 | 1:1000 (IF) |
| Anti-Rabbit IgG Alexa Fluor 488 | Donkey | Abcam ab150073 | 1:1000 (IF) |
| Anti-Mouse IgG Alexa Fluor 594 | Goat | Abcam ab150116 | 1:1000 (IF) |
| Anti-Mouse IgG HRP | Donkey | Jackson ImmunoResearch 715-035-151 | 1:5000 (Wes) |
| Anti-Rabbit IgG HRP | Donkey | Jackson ImmunoResearch 711-035-152 | 1:5000 (Wes) |
| Anti-Rat IgG HRP | Goat | Invitrogen 31470 | 1:5000 (Wes) |
| Vinculin | Rabbit | Abcam ab91459 | 1:1000 (Wes) |
| NeuN | Mouse | Millipore MAB377 | 1:500 (IF, Wes) |
| GFAP | Rat | Invitrogen 13-0300 | 1:1000 (IF, Wes) |
| Iba1 | Rabbit | WAKO 019-19741 | 1:500 (IF, Wes) |
| GFP | Rabbit | Invitrogen A-11122 | 1:1000 (IF) |
| mEM48 | Mouse | Millipore MAB5374 | 1:50 (IF, Wes) |
| Anti-Polyglutamine  -Expansion Diseases Marker(1C2) | Mouse | Millipore MAB1574 | 1:1000 (Wes) |
| Flag | Mouse | Millipore F1804 | 1:1000 (Wes) |
| GAPDH | Mouse | Abcam ab8245 | 1:1000 (Wes) |

Supplementary Table 4. Information of the pigs used in this manuscript.

| Name | Genetic background | Sex | Age to treat | Age to analyze |
| --- | --- | --- | --- | --- |
| F4-56 | HD | M | 3M | 7M |
| F4-51 | HD | F | 3M | 7M |
| F4-59 | HD | M | 3M | 7M |
| F4-61 | HD | F | 3M | 7M |
| F4-62 | HD | F | 3M | 7M |
| F4-70 | HD | F | 3M | 7M |
| F4-66 | HD | M | 3M | 7M |
| F4-65 | HD | M | 3M | 7M |
| WT-1 | WT | M | 3M | 7M |
| WT-2 | WT | F | 3M | 7M |
| WT-3 | WT | F | 3M | 7M |
| WT-4 | WT | M | 3M | 7M |
